# Wildfire-related smoke inhalation worsens cardiovascular risk in sleep disrupted rats

**DOI:** 10.3389/fenvh.2023.1166918

**Published:** 2023-05-30

**Authors:** W. Kyle Martin, M. C. Schladweiler, W. Oshiro, J. Smoot, A. Fisher, W. Williams, M. Valdez, C. N. Miller, T. W. Jackson, D. Freeborn, Y. H. Kim, D. Davies, M. Ian Gilmour, U. Kodavanti, P. Kodavanti, M. S. Hazari, A. K. Farraj

**Affiliations:** 1 Curriculum in Toxicology and Environmental Medicine, UNC, Chapel Hill, NC, United States; 2 Public Health & Integrated Toxicology Division, US EPA, Research Triangle Park, NC, United States; 3 Oak Ridge Institute for Science and Education, Oak Ridge, TN, United States

**Keywords:** cardiovascular risk, air pollution, wildfires, eucalyptus smoke, sleep

## Abstract

**Introduction:**

As a lifestyle factor, poor sleep status is associated with increased cardiovascular morbidity and mortality and may be influenced by environmental stressors, including air pollution.

**Methods:**

To determine whether exposure to air pollution modified cardiovascular effects of sleep disruption, we evaluated the effects of single or repeated (twice/wk for 4 wks) inhalation exposure to eucalyptus wood smoke (ES; 964 μg/m^3^ for 1 h), a key wildland fire air pollution source, on mild sleep loss in the form of gentle handling in rats. Blood pressure (BP) radiotelemetry and echocardiography were evaluated along with assessments of lung and systemic inflammation, cardiac and hypothalamic gene expression, and heart rate variability (HRV), a measure of cardiac autonomic tone.

**Results and Discussion:**

GH alone disrupted sleep, as evidenced by active period-like locomotor activity, and increases in BP, heart rate (HR), and hypothalamic expression of the circadian gene *Per2*. A single bout of sleep disruption and ES, but neither alone, increased HR and BP as rats transitioned into their active period, a period aligned with a critical early morning window for stroke risk in humans. These responses were immediately preceded by reduced HRV, indicating increased cardiac sympathetic tone. In addition, only sleep disrupted rats exposed to ES had increased HR and BP during the final sleep disruption period. These rats also had increased cardiac output and cardiac expression of genes related to adrenergic function, and regulation of vasoconstriction and systemic blood pressure one day after final ES exposure. There was little evidence of lung or systemic inflammation, except for increases in serum LDL cholesterol and alanine aminotransferase. These results suggest that inhaled air pollution increases sleep perturbation-related cardiovascular risk, potentially in part by increased sympathetic activity.

## 1. Introduction

Cardiovascular disease is the preeminent cause of premature mortality and morbidity worldwide (1). Many lifestyle and environmental factors are implicated in the instigation and progression of cardiovascular disease, such as exposure to toxic agents (e.g., inhaled air pollution), medication side effects, and negative maintenance of health through poor diet, sedentary lifestyle, and inadequate sleep. Poor sleep is a burgeoning global public health problem exacerbated by psychosocial stress associated with socioeconomic disadvantage (2, 3), climate change (4), the increased prevalence of wildfires (5, 6), and incompletely understood emerging threats such as the COVID-19 pandemic (7), among other factors. Perturbations in sleep patterns, including insomnia or disrupted sleep, alter cardiovascular function and increase risk of atherosclerosis, stroke, and hypertension (8–10). Both acute and chronic sleeplessness have been linked to increased stress hormone release, impaired nocturnal blood pressure dipping, autonomic imbalance, endothelial dysfunction, oxidative stress, inflammation, and increased insulin resistance (11–16).

Emerging reports indicate that environmental factors can aggravate sleeplessness-related morbidity. For example, in patients with obstructive sleep apnea, outdoor PM_2.5_ was associated with increased systemic inflammatory markers (17), and, separately, increases in bedroom PM_10_ were associated with more severe disease (18). Disrupted sleep has also been shown to interact with ambient PM pollution to worsen cardiac conduction responses in the middle-aged and elderly (19). Wildland fires, which represent a major source of PM, are increasingly linked to adverse health impacts from inhaled smoke (20), and are projected to increase in frequency and severity in the coming decades (21). The widespread and detrimental effects of inadequate sleep may impart an underlying vulnerability that serves as a substrate for increased responsiveness to inhaled air pollution, including wildland fire smoke. Human studies exploring this relationship typically rely on self-reported sleep status and/or unintentional inhalation of ambient air pollution; consequentially, the interactive effects of sleep disruption and air pollution exposure and the associated mechanisms driving responses remain poorly characterized.

The purpose of this study was to determine the impacts of controlled exposure to eucalyptus smoke (ES), a key contributor to wildland fire air pollution that has been linked to adverse cardiopulmonary outcomes (22, 23), on sleep disruption-related cardiovascular responses. We hypothesized that exposure to wildland fire-related air pollution would exacerbate sleep disruption-induced cardiovascular pathophysiology. To examine this hypothesis, we adapted a model of sleep loss (24–26) in which rats undergo sleep disruption *via* brief, gentle handling once every 30 min for 5 h during their inactive period/sleep window. We first demonstrated the utility of this sleep loss model in unexposed rats by characterizing locomotor activity, used to confirm wakefulness, and cardiovascular responses using implantable telemetry. Second, in a separate cohort of implanted rats, we examined the interactive effects of poor sleep and air pollution exposure by disrupting sleep 1 h prior to each of eight total exposures to ES over the course of four weeks. Blood pressure, the electrocardiogram, heart rate, and locomotor activity were assessed before and after each sleep disruption and exposure period and were related to heart rate variability to determine linkages with altered cardiac autonomic tone. Cardiovascular function and dimensions, pulmonary and systemic markers of injury and inflammation, and cardiac and hypothalamic gene expression were also measured.

## 2. Materials and methods

### 2.1. Animals

Ten-to-twelve-week-old, male Sprague Dawley rats were housed 1/cage in polycarbonate cages and maintained on a 12 h light/dark cycle at approximately 22°C and 50% relative humidity in our Association for Assessment and Accreditation of Laboratory Animal Care-approved facility and acclimated for a minimum of one week before exposures. All animals received standard (5,001) Purina pellet rat chow (Brentwood, MO) and water *ad libitum*. The Institutional Animal Care and Use Committee of the U.S. Environmental Protection Agency (U.S. EPA) approved all protocols. All animal experiments complied with the ARRIVE guidelines and were carried out in accordance with the National Research Council’s Guide for the Care and Use of Laboratory Animals (27).

### 2.2. Experimental design and group size determinations

**FIGURE 1:**
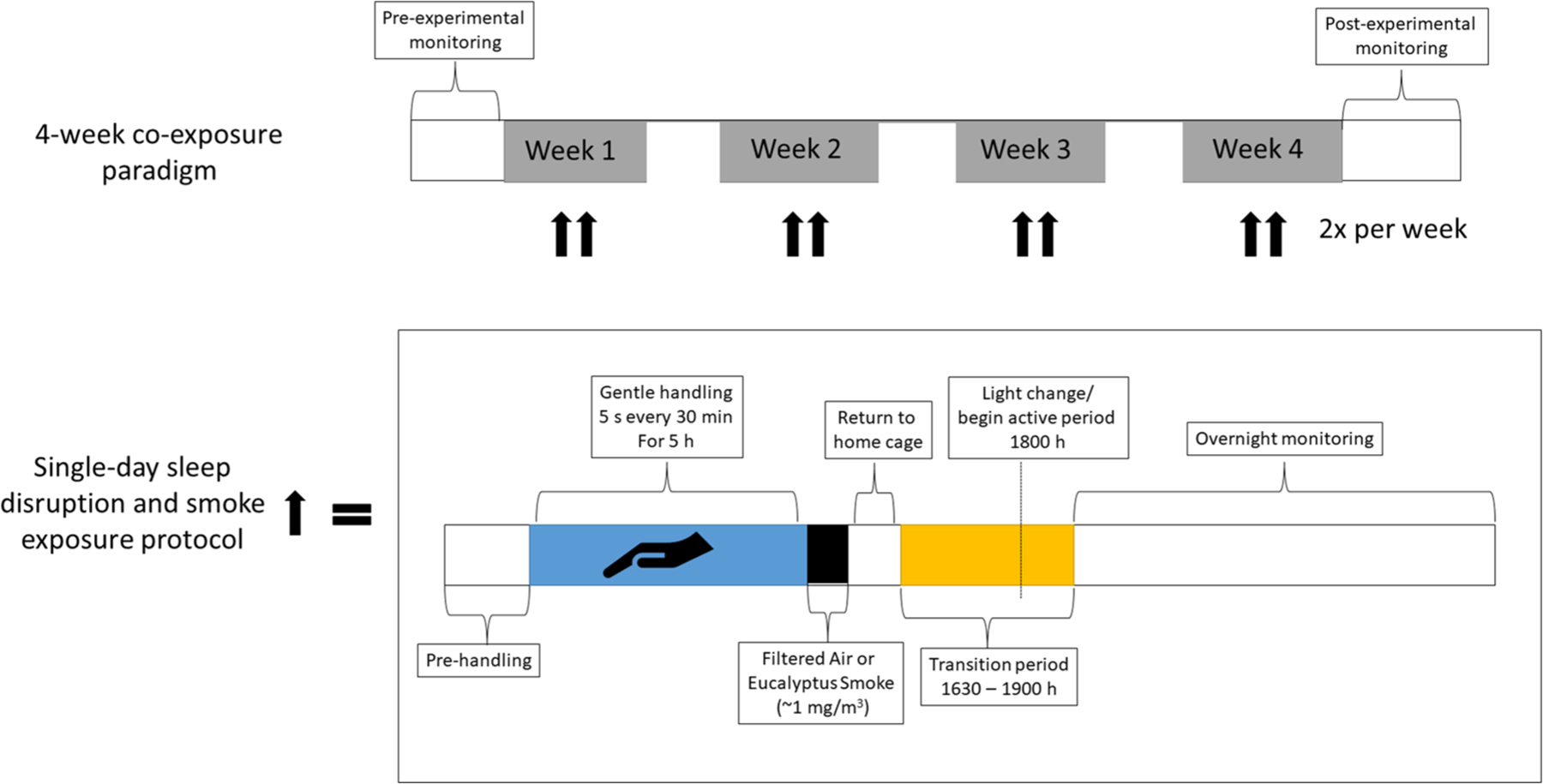
Timeline of sleep disruption and ES exposures. Rats underwent either one day or eight days (twice per week for four weeks) of sleep disruption and exposure to ES each day, rats were sleep disrupted by gentle handling for 5 h (~9 AM to 2 PM) during their normal “rest” period and then exposed for 1h (~3 pm to 4 pm) to ES (964 μg/m^3^) or FA. A subset of rats was implanted with telemeters to allow for continuous monitoring of activity, a surrogate for wakefulness, as well as blood pressure, heart rate, the electrocardiogram, and body temperature throughout the entire regimen.

There were two studies conducted: Study 1, which characterized the physiological impacts of a single rodent handling (i.e., sleep disruption) in un-exposed rats and Study 2, which assessed the impacts of sleep disruption caused by rodent handling on cardiopulmonary responses to ES over the course of 4 weeks for a total of eight co-exposures. Study 1 consisted of three groups of rats (*n* = 8/group): (1) rats implanted with radiotelemeters to monitor activity and cardiovascular function, (2) rats that were euthanized immediately after the rodent handling protocol to assess immediate impacts on stress markers, and (3) rats that were euthanized one day after rodent handling to assess delayed impacts on stress markers. Study 2 consisted of three cohorts of rats (*n* = 7 or 8/group), each of which had four groups of rats: (1) Normal Sleep-Filtered Air (NSFA), (2) Normal Sleep-Air Pollution (NSAP), (3) Sleep Disruption-Filtered Air (SDFA), and (4) Sleep Disruption-Air Pollution (SDAP). Cohort 1 was euthanized one day after a single bout of sleep disruption and ES exposure and was used to assess biological/tissue responses after acute sleep disrupution/ES exposure; Cohort 2 was implanted with telemeters to monitor activity and cardiovascular function and underwent sleep disruption followed by exposure to ES or filtered air (FA) twice per week for four weeks; Cohort 3 was sleep disrupted and exposed to ES or FA twice per week for four weeks and underwent cardiovascular ultrasound three days after the final exposure to assess impacts on cardiovascular function and dimensions and was euthanized two days after ultrasound to assess biological/tissue responses (Figure 1 and Supplementary Table S1).

Group size for Study 2 was based on effect range and standard deviation (SD) determinations from Study 1 blood pressure data. Sample size analysis was performed using open source R Studio software with the “pwr”package and “pwr.anova.test” command (https://cran.rproject.org/web/packages/pwr/pwr.pdf). Sample size analysis was based upon the (k) number of experimental groups (*k* = 4 groups), a significance level = 0.05, a power = 0.8, and the effect size index (f), which is derived by multiplying the expected effect size (d) by the standard deviation (SD).

### 2.3. Telemeter implantation

Animals were anesthetized with ketamine/xylazine (80 mg/ml ketamine HCL and 12 mg/ml xylazine HCL; Sigma Chemical Co., St. Louis, MO), and implanted with radio-telemeters that transmit electrocardiogram, heart rate, aortic blood pressure, locomotor activity and core body temperature (model HD-S11, Data Sciences International, St. Paul, MN) at Charles River Laboratories as described previously (28).

### 2.4. Radiotelemetry of physiological parameters in Study 2 Cohort 2 rats

Radiotelemetry was used to monitor BP, HR, the electrocardiogram, and activity in conscious, unrestrained rats. Figure 1 illustrates a timeline of exposure and cardiovascular monitoring using telemetry. Data were recorded for 3 min every 10 min in their home cages before, during, and after sleep disruption and after ES exposure. Arterial BP (systolic and diastolic pressure and mean arterial pressure) and HR were calculated automatically by software [Ponemah; Data Sciences International, Inc (RRID:SCR_017107)] from pressure and ECG waveforms sampled at 1,000 Hz. HRV analysis was analyzed using the same software and generated HR and time-domain measures, including mean time between adjacent QRS-complex peaks (RR interval), standard deviation of the RR interval (SDNN), square root of the mean of squared differences of adjacent RR intervals (RMSSD), and percent of adjacent normal RR intervals differing by ≥50 ms (pNN50). HRV analysis also provided frequency-domain parameters, including low frequency (LF: 0.200–0.750 Hz) and high frequency (HF: 0.750–2.00 Hz), and the ratio of these two frequency domains (LF/HF). For frequency-domain analysis, the signal was analyzed with a Hamming window for segment lengths of 512 samples with 50% overlapping. Within Ponemah, the settings for variability analysis in the frequency domain were VLF (0.05–0.25), LF (0.25–1), HF (1–3) for the “rat bin set”. A quadratic interpolation was set at a rate of 50 Hz and a tolerance of 20 ms. Calculations were made using a Hamming windowing method with a segment duration of 30 s and a frequency axis range of 0–5.0 Hz. For the time domain the reporting period was set to 5 min.

### 2.5. Acclimation to exposure chambers and ES concentrations

All animals were acclimated three times in 10 min increments to full-body inhalation chambers at room temperature. Final acclimations occurred at least 24 h before exposure. We used eucalyptus wood which was purchased as writing pen blanks (rectangles at 0.75 inches square by 6 in long; Woodworkers Source Arizona). Eucalyptus is present worldwide and its combustion is responsible for substantial portions of particulate matter from wildland fire smoke in Australia and coastal California, USA (29) as well as, increasingly, the US Southeast (30). This wood was processed through a gasoline powered wood shredder (Echo bearcat model number SC3206). The shredder was cleaned between uses so no cross contamination would occur. Animals were exposed once for a duration of 1 h to ES generated using an automated control tube furnace system. The target ES concentration based on fine particulate matter (PM_2.5_; ≤2.5 microns in aerodynamic diameter) concentration was ~1.0 mg/m^3^ (Table 1).

### 2.6. Tube furnace exposure system & PM and gas monitoring and sampling

Flaming ES was generated using an automated quartz-tube furnace system. An automated mass flow controller (Mass-Flo, MKS Instrument, Inc., Andover, MA) based on a proportional-integral-derivative (PID) feedback loop was incorporated into the system to precisely control smoke concentration. ES (2 L/min) generated from the tube furnace system was diluted with FA (FA; ~3 and ~60 L/min for 1st and 2nd dilution air, respectively) and then delivered to a whole-body inhalation chamber as previously described (31). The smoke concentration was monitored continuously and adjusted by the PID feedback control loop linked to a continuous PM monitor in the chamber paired to an exhaust flow control valve in a smoke inlet line. The adjustment was made immediately as soon as a change in PM concentration in the chamber was detected to maintain PM concentration close to its set point (<10% of the target set point). The ES in the chamber was maintained at a temperature of ~72°F, and ~40% relative humidity, controlled by a humidifier. A pressure gauge (Magnehelic, Dwyer Instruments Inc., Michigan City, IN) was placed in the chamber to ensure constant pressure throughout the inhalation exposure. We monitored carbon dioxide (CO2) and carbon monoxide (CO) levels using a non-dispersive infrared analyzer (Model: 602 CO/CO2; CAI Inc., Orange, CA) and nitrogen oxides (NO, NO2, and NOx) using a chemiluminescent analyzer (Model: 42i NO/NO2/NOx; Thermo Scientific, Franklin, MA). We also collected PM on a glass-fiber filter installed in an exhaust line of the inhalation chamber to determine mean PM concentrations gravimetrically by weighing the filter before and after inhalation exposure. The real-time measurements of ES properties and engineering parameters (e.g., temperature, RH, static pressure, and flow rate) were monitored continuously, recorded, and displayed using data acquisition software (Dasylab version 13.0, National Instruments, Austin, TX).

### 2.7. Ultrasound echocardiography and analysis in Study 2 Cohort 3 rats

Cardiac function of animals in Cohort 3 was determined at baseline and 3 days after the final exposure using a high frequency echocardiography ultrasound system [Vevo 2100, FujiFilm Visual Sonics Inc., Toronto, Canada (RRID:SCR_015816)] as described previously (22). An MS-201 transducer was used to record 3 video loops of the parasternal long axis views of the left ventricle in M-mode (15 MHz) noninvasively for functional measurements. The sonographer was blinded to treatment group identities. Echocardiography data analyses were also performed while blinded to identities of exposure groups using Vevo^®^ LAB software (FujiFilm VisualSonics Inc., Toronto, Canada). Two beats between breaths from each of the three cine loops were collected. This yielded a total of six beats analyzed per animal. Long-axis M-mode loops were used to determine heart rate, stroke volume (SV), cardiac output (CO), ejection fraction (EF), fractional shortening, end systolic volume (ESV), end diastolic volume (EDV), and left ventricular anterior and posterior wall thickness (LVAW and LVPW).

### 2.8. Necropsy & serum and plasma collection

Animals in Study 1 and Study 2 were euthanized *via* intraperitoneal injection of 1 ml/kg pentobarbital (Fatal-Plus, Dearborn, MI) diluted 1:1 approximating 200 mg/ml. When animals were completely non-responsive to hind paw pinch, blood was collected through the abdominal aorta in serum separator tubes and EDTA tubes, which were inverted and placed on ice. Before spinning the EDTA tubes, complete blood cell counts (CBC) were determined. Red blood cells (RBC), white blood cells (WBCs), red cell distribution width (RDW%), hematocrit (HCT), mean corpuscular volume (MCV), hemoglobin (HB), mean cell hemoglobin (MCH), mean corpuscular hemoglobin concentration (MCHC), platelets (PLT), plateletcrit (PCT) and mean platelet volume (MPV) were measured utilizing a Beckman-Coulter AcT blood analyzer (Beckman-Coulter Inc., Fullerton, CA). The serum and EDTA tubes were then centrifuged at 1500 g for 10 min and serum and plasma samples were stored at −80°C until further analysis.

### 2.9. Measures of systemic markers and hormones in Study 1 and Study 2

Only corticosterone (Arbor Assays, Ann Arbor, MI) was measured in the plasma of Study 1 rats. In Study 2 rats, several serum factors were measured using the Konelab Arena 30 Clinical Chemistry Analyzer (Thermo Clinical LabSystems, Espoo, Finland) including total cholesterol (TC), triglycerides (TG), creatine kinase (CK), alkaline phosphatase (AP), glucose (TECO Diagnostics, Anaheim, CA), high-density lipoprotein (HDL), low-density lipoprotein (LDL), and total cholesterol (Sekisui Diagnostics, PE, Canada), free fatty acids (FFA; Cell Biolabs, Inc., San Diego, CA), fibrinogen, D-dimer, C3 and C4 (Kamiya Biomedical, Seattle, WA), alanine amino-transferase (ALT) and angiotensin-converting enzyme (ACE) (Thermo Scientific, Middletown, VA). Serum fatty acid binding protein (FABP) and myosin light chain 3 (Myl3) (Meso Scale Discovery, Rockville, MD) were also measured in the serum of Study 2 rats.

### 2.10. Bronchoalveolar lavage fluid (BALF) collection and analysis

BALF samples were processed to determine total cell counts and cell differentials (Z1 Beckman-Coulter Counter, Miami, FL). Cell-free BALF samples were used to measure markers of injury using the Konelab Clinical Analyzer. Separate kits were used to measure total protein (Coomassie Plus Protein Assay Kit; Pierce Biotechnology, Inc., Rockford, IL), albumin (Sekisui Diagnostics, PE, Canada), lactate dehydrogenase (LDH) activity, γ-glutamyl transferase (GGT) activity (Thermo Fisher Diagnostics, Middletown, VA), and b-N-Acetylglucosaminidase (NAG) activity (Sigma-Aldrich Diagnostics St. Louis, MO).

### 2.11. RNA isolation, gene expression analysis and data analysis in cardiac left ventricle and hypothalamus tissue

Total RNA was isolated from the left ventricle of the heart and the hypothalamus using Trizol reagent (Invitrogen) following a phenol-chloroform extraction protocol. RNA concentrations and integrity were confirmed using a NanoDrop spectrophotometer (Thermo Scientific, Wilmington, DE) and Qubit fluorometer BR (Broad Range) assay kit (Thermofisher) prior to shipment to Nanostring for gene expression quantification using their nCounter Analysis System. The genes assessed include those related to excitation, contraction, inflammation, and oxidative stress, among others in the heart (a total of 98 genes) and circadian rhythm and oxidative stress and inflammation, among others, in the hypothalamus (a total of 77 genes). This system is based on direct multiplexed quantification of nucleic acids enabling the profiling of hundreds of unique target mRNAs in a single reaction. Gene expression was quantified using nSolver Analysis Software (RRID:SCR_003420), an integrated analysis platform for storage, custom QC, and custom normalization of nCounter data. Data was analyzed with a HyperScale architecture by ROSALIND, Inc. (San Diego, CA). Read Distribution percentages, violin plots, identity heatmaps, and sample MDS plots were generated as part of the QC step. Normalization, fold changes and *p*-values were calculated using criteria provided by Nanostring. ROSALIND^®^ follows the nCounter^®^ Advanced Analysis protocol of dividing counts within a lane by the geometric mean of the normalizer probes from the same lane. Housekeeping probes used for normalization (i.e., actin beta (ACTB), beta-2-microglobulin (B2M), and glyceraldehyde-3-phosphate dehydrogenase (GAPDH)) were selected based on the geNorm algorithm as implemented in the NormqPCR R library1 (RRID:SCR_003388). Abundance of various cell populations is calculated on ROSALIND using the Nanostring Cell Type Profiling Module. Fold changes and *p*-values are calculated using the fast method as described in the nCounter^®^ Advanced Analysis 2.0 User Manual. *P*-value adjustment was performed using the Benjamini-Hochberg method of estimating false discovery rates (*p* < 0.05). Group differences in expression levels were considered significantly different compared to the control group if the fold changes were 1.25-fold or greater and the *p*-value, corrected for multiple comparisons, was <0.05. Hypergeometric distribution was used for enrichment analysis for gene ontology. The topGO R library (RRID:SCR_014798) was used to determine local similarities and dependencies between GO terms in order to perform Elim pruning correction.

### 2.12. Statistics

Data are reported as boxplots with all data points shown. Box edges mark the interquartile range, middle line marks the median, the “+” marks the mean, and the whiskers mark the minimum and maximum data values. GraphPad Prism [GraphPad Software version 7.02, San Diego, CA (RRID:SCR_002798)] was used for all statistical analyses. Normality of data distributions was assessed with normality tests (D’Agostino-Pearson omnibus test or, in the instances of smaller “n,” Shapiro-Wilk or Kolmogorov-Smirnov tests) with significance set at *p* < 0.05. All non-physiology data that met this assumption were assessed using a one-way or two-way ANOVA with Tukey’s posttest for multiple comparisons. Data that did not meet the normality assumption were tested using the nonparametric Kruskal-Wallis test with Dunn’s multiple comparisons posttest. A *p*–value <0.05 was considered statistically significant. Functional values (i.e., telemetry and echocardiography endpoints) were assessed with repeated measures two-way ANOVA with Tukey’s posttest and multiplicity-adjusted *p*-values with *p* values <0.05 considered statistically significant.

HRV data were analyzed statistically in R v4.1.3 (https://www.R-project.org/). For each endpoint, normality was assessed using the Shapiro-Wilk test. If any group failed to meet normality, data were transformed using the box cox transformation and assessed subsequently for normality. Data were examined to confirm that no individual had more than 15% missing data in the time domain (frequency domain data did have higher loss as it is more susceptible to noise interference), and any missing data were imputed using predictive mean matching. Missing data were a consequence of noise in the signal and Supplementary Table S2 shows the percentage of missing and imputed data for each parameter. Linear modeling was performed using the afex package (RRID:SCR_022857) with two between-subjects factors (sleep, AP exposure) and one within-subjects factor (time). Type III contrasts were performed using the emmeans (RRID: SCR_018734) package with pairwise comparisons, and homogeneity of variance was assessed using Levene’s test. Outcomes of statistics tests that are used to support conclusions in this manuscript are included as a Supplementary Excel document.

## 3. Results

### 3.1. Characterization of ES

Gas and particle concentrations for all exposures are indicated in Table 1. The average PM_2.5_ mass concentration for all exposures was 0.964 mg/m^3^. The average particle size was 180 nm ± 1.33.

### 3.2. Characterization of gentle handling model in Studies 1 and 2

During the resting period, gentle handling significantly increased average activity, heart rate, body temperature, and systolic and diastolic blood pressure relative to the unhandled group in Study 1 (Supplementary Figure S1 shows averages over the 5 h period). There were no statistically significant changes in plasma corticosterone immediately or one day after handling (Supplementary Figure S2). These functional cardiovascular results were reproduced during the handling window of Day 1 of Study 2 in normal sleep and sleep disrupted rats prior to air pollution exposure (Supplementary Figure S3 shows changes averaged over 5 h in normal sleep and sleep disrupted rats). When all rats were binned into two groups, sleep disrupted rats had significantly greater activity, heart rate, body temperature, and systolic and diastolic blood pressure. When split into the final 4 groupings (Note: Normal-Group 1, Normal-Group-2, Sleep-disrupted Group-1, and Sleep-Disrupted Group 2 eventually became Normal Sleep-Filtered Air, Normal Sleep-Air Pollution, Sleep-Disrupted-Filtered Air and Sleep-Disrupted-Air Pollution, respectively, after air pollution exposure) activity and heart rate increases remained statistically significant, whereas the differences in the blood pressure parameters were no longer significantly different prior to air pollution exposure. Supplementary Figure S4 shows changes over time measured every 10 min over the course of the 5 h period in the four groups prior to ES exposure. As evident in Supplementary Figure S4, activity, heart rate and blood pressure responses in the sleep disrupted groups spike immediately at the beginning of the handling period and gradually return to levels on par with the undisrupted groups by the end of the handling period. Activity, heart rate and blood pressures responses were similar among the two sleep disrupted groups.

### 3.3. Heart rate and blood pressure during the transition to the active period after exposure to ES on Day 1 of Study 2

**FIGURE 2:**
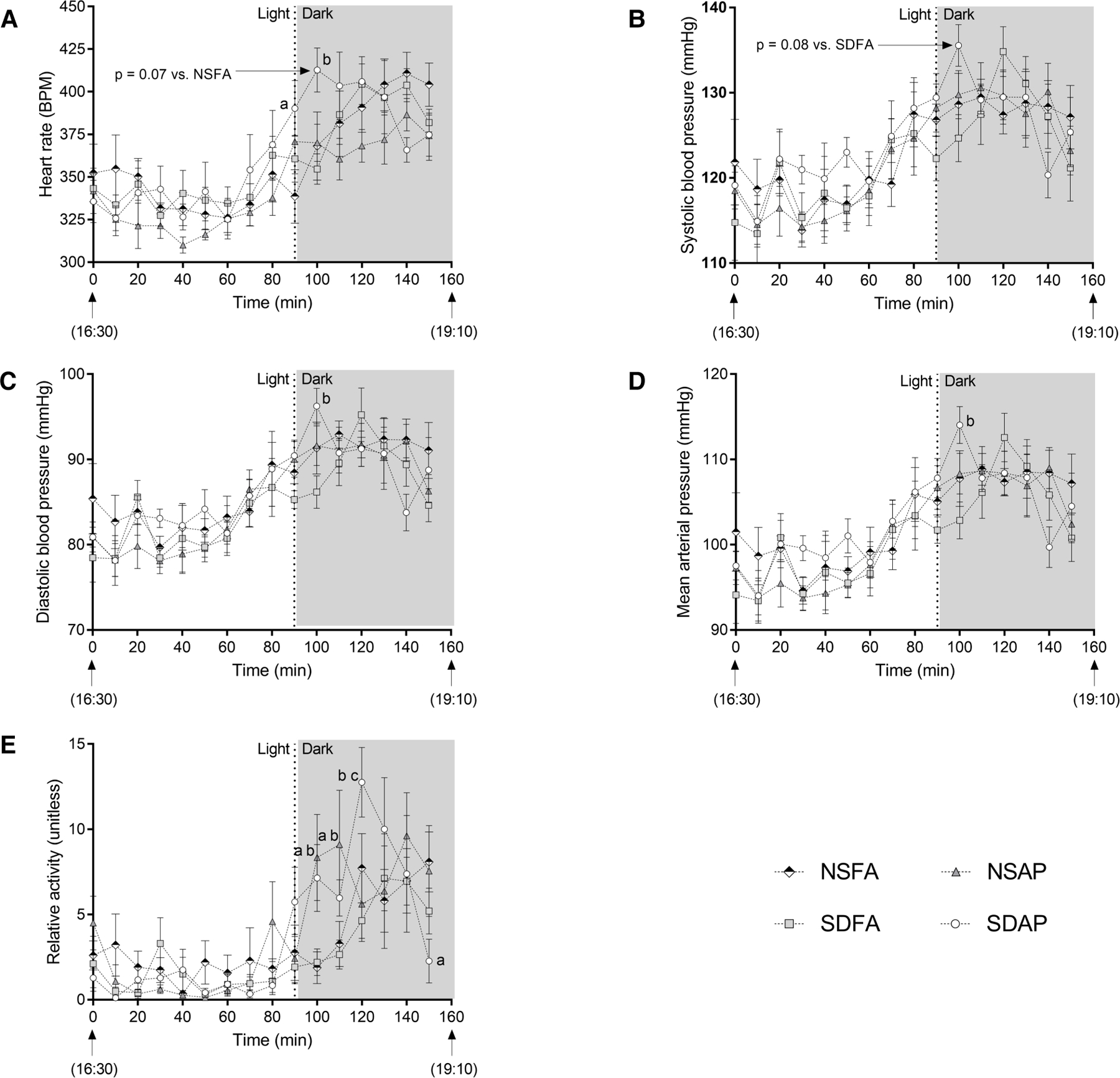
Cardiovascular responses before and after the transition from light to dark immediately after exposure to ES or FA on Day 1. Heart rate (**A**), systolic (**B**) and diastolic (**C**) blood pressure, mean arterial pressure (**D**) and activity (**E**) were recorded while rats were in their home cages approximately 90 min before and 70 min after the light change. Data represent averages of 3 min of data (*n* = 7–8) recorded every 10 min in normal rats exposed to filtered air (NSFA) or ES air pollution (NSAP) and sleep-disrupted rats exposed to filtered air (SDFA) or ES air pollution (SDAP). a - significantly different from NSFA (*p* < 0.05). b - significantly different from SDFA (*p* < 0.05). c - significantly different from NSAP (*p* < 0.05). *P*-values for tendencies towards significant changes are also indicated.

There was a significant increase in heart rate in SDAP relative to NSFA at the light change (i.e., lights off). In the subsequent 10 min period, SDAP had significantly greater heart rate than SDFA (Figure 2A) and tendencies towards significantly greater heart rate than NSFA (*p* = 0.07) and systolic blood pressure than SDFA [*p* = 0.08; (Figure 2B)]. SDAP also had significantly greater diastolic blood pressure and mean arterial pressure than SDFA (Figures 2C, D) during this period and a tendency towards significantly greater activity than NSFA (*p* = 0.07; Figure 2E). NSAP also had significantly greater activity than NSFA. There were no significant differences among the groups in any parameters in the transition period one day after exposure (Supplementary Figure S5).

### 3.4. Heart rate variability during the transition to the active period after exposure to ES on Day 1 of Study 2

**FIGURE 3:**
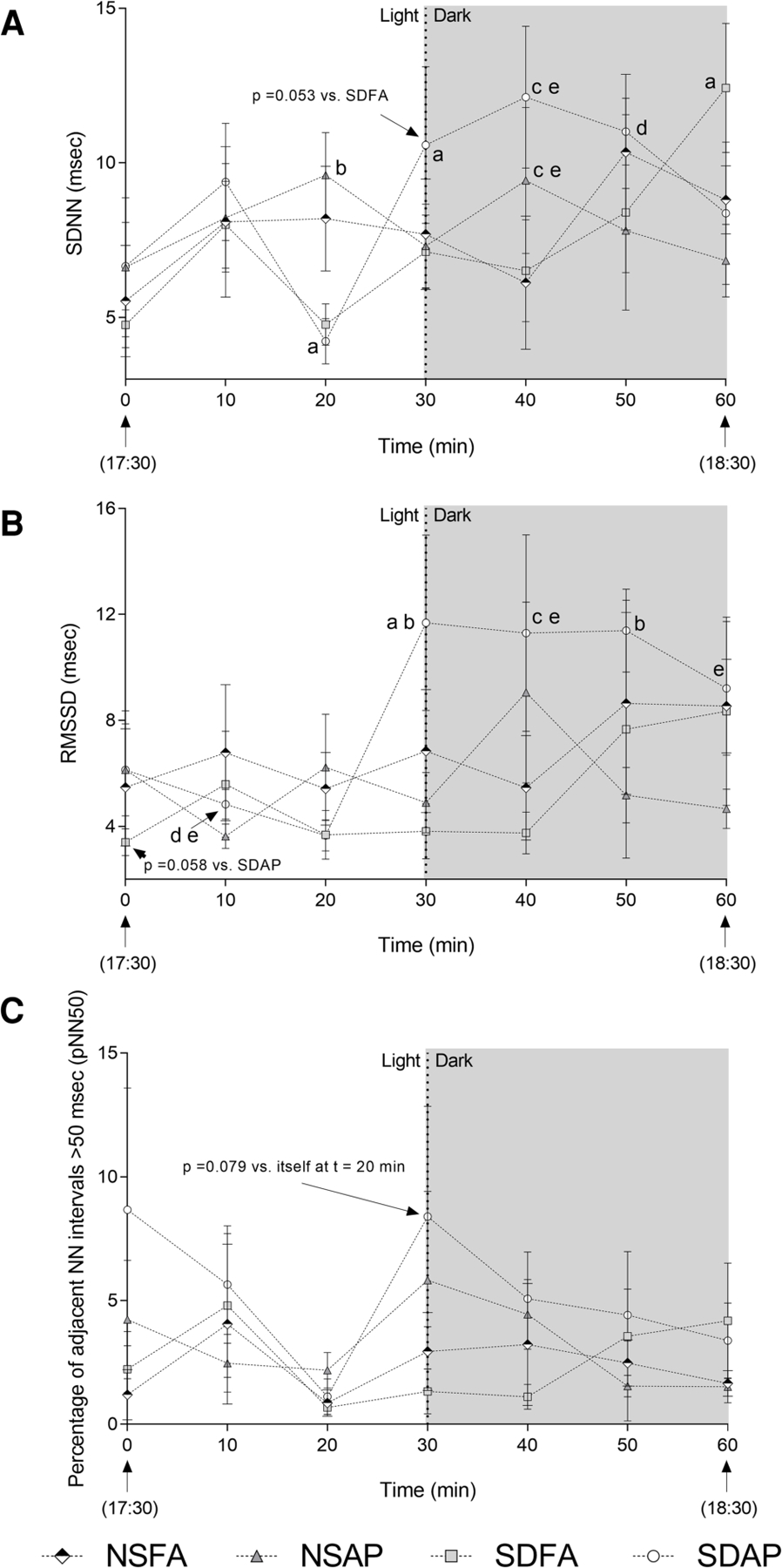
Time domain heart rate variability responses before and after the transition from light to dark immediately after exposure to ES or FA on Day 1. Standard deviation of the normal–normal RR interval (SDNN; **A**), root mean square of the standard deviation of the normal–normal RR interval (RMSSD; **B**), and the percent of adjacent normal RR intervals differing by ≥50 ms (pNN50; **C**) were recorded while rats were in their home cages approximately 30 min before and 30 min after the light change. Data represent averages of 3 min of data (*n* = 7–8) recorded every 10 min in normal rats exposed to filtered air (NSFA) or eucalyptus smoke air pollution (NSAP) and sleep-disrupted rats exposed to filtered air (SDFA) or eucalyptus smoke air pollution (SDAP). a - significantly different from itself during the immediately preceding time period (*p* < 0.05). b – significantly different than all other groups within time period (*p* < 0.05). c – significantly different from NSFA within time period (*p* < 0.05). d – significantly different than NSAP within time period (*p* < 0.05). e – significantly different than SDFA (*p* < 0.05) within time period.

To determine if the heart rate and blood pressure responses that took place during the transition from light to dark were accompanied by changes in autonomic tone, HRV was assessed beginning 30 min before the transition and extending until 30 min after. SDAP had a significant decrease in SDNN (Figure 3A) in the immediate period before the light change (i.e., *t* = 20 min) relative to itself in the immediately preceding period (i.e., *t* = 10 min). SDAP, SDFA and NSFA all had significantly lower SDNN relative to NSAP at *t* = 20 min. Only SDAP had significant increases in SDNN (Figure 3A) and RMSSD (Figure 3B) and a tendency towards increased pNN50 (*p* = 0.078; Figure 3C) at the time of the light change (i.e., *t* = 30 min) relative to itself in the period immediately before the light change (i.e., *t* = 30 min). SDAP had a tendency towards greater SDNN relative to SDFA (*p* = 0.053) and significantly greater RMSSD relative to NSFA, NSAP, and SDFA at *t* = 30 min. SDAP and NSAP had significantly greater SDNN relative to NSFA and SDFA and SDAP had significantly greater RMSDD relative to NSFA and SDFA at *t* = 40 min. SDAP had significantly greater SDNN relative to SDFA and had significantly greater RMSDD relative to NSFA, NSAP, and SDFA at *t* = 50 min. SDAP had a significant increase in SDNN at *t* = 60 min relative to itself at *t* = 50 min. SDAP also had significantly greater RMSSD relative to SDFA at *t* = 60 min. There were little-to-no changes in frequency domain parameters (Supplementary Figure S6).

### 3.5. Heart rate and blood pressure responses during the handling window just before the final exposure in Study 2

**FIGURE 4:**
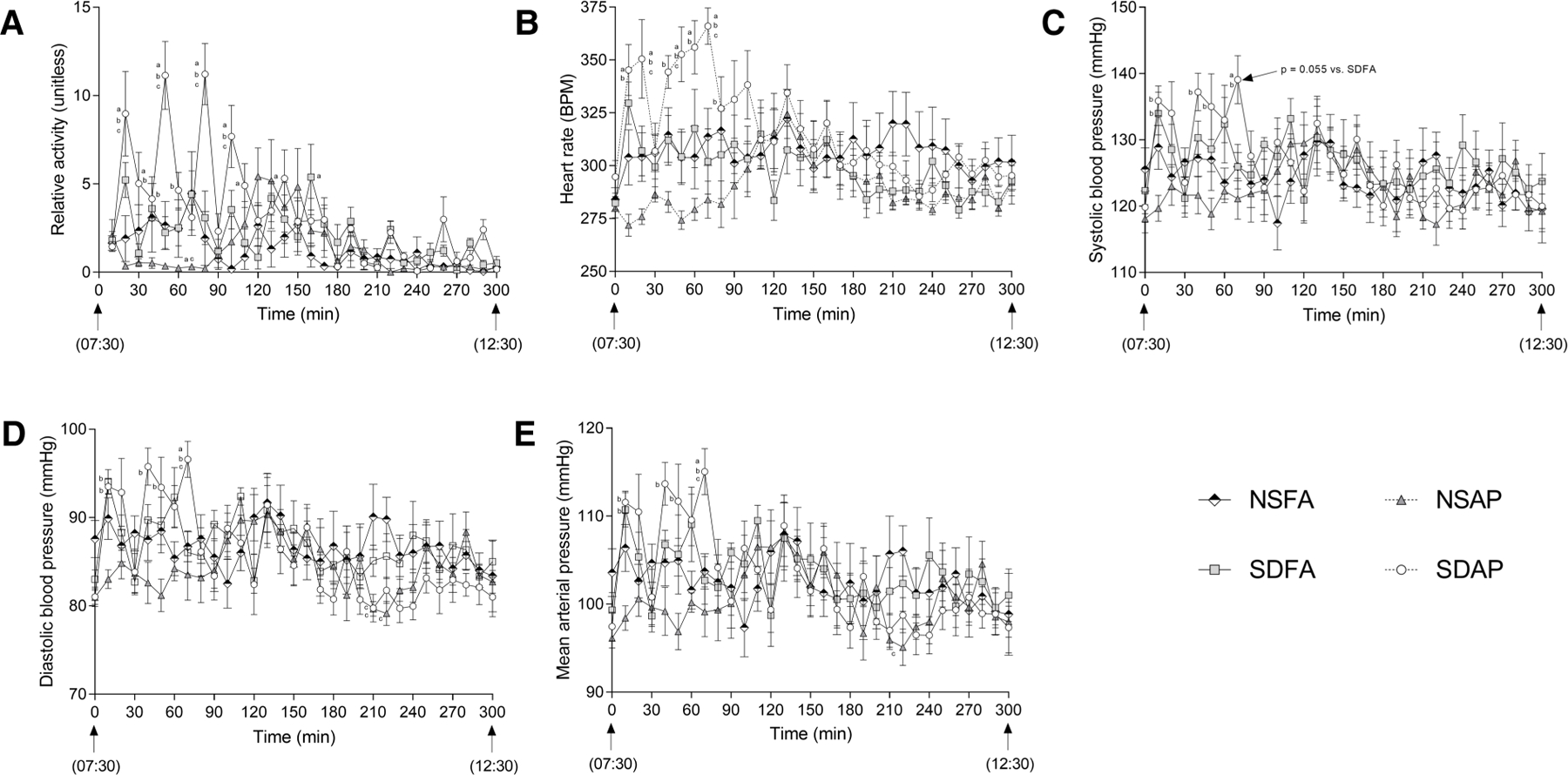
Cardiovascular responses and activity during rodent handling on Day 8. Activity (**A**), heart rate (**B**), systolic (**C**) and diastolic (**D**) blood pressure (BP), and mean arterial pressure (**E**) were recorded during the entire 5h handling period. Data represent averages of 3 min of data (*n* = 7–8) recorded every 10 min in normal rats exposed to filtered air (NSFA) or eucalyptus smoke air pollution (NSAP) and sleep-disrupted rats exposed to filtered air (SDFA) or eucalyptus smoke air pollution (SDAP). a - significantly different from NSFA (*p* < 0.05). b - significantly different from NSAP (*p* < 0.05). c - significantly different from SDFA (*p* < 0.05). p-values for tendencies towards significant changes are also indicated.

During the Day 8 handling window, SDAP had significantly greater activity (Figure 4A) and heart rate (Figure 4B) than all remaining groups at multiple time points during the early part of the handling window. At 70 min into the handling window, SDAP had significantly higher diastolic blood pressure (Figure 4D) and mean arterial pressure (Figure 4E) than all other groups, and significantly higher systolic blood pressure (Figure 4C) than NSAP and NSFA and a tendency towards higher blood pressure than SDFA ( *p* = 0.055). There were little-to-no changes in any of the parameters after this period.

### 3.6. Heart rate variability during the handling window just before the final exposure of Study 2

During the Day 8 handling window, the were a handful of instances where there were group differences within specific timepoints (Supplementary Figures S7, S8). These were mostly among both sleep disrupted groups irrespective of exposure.

### 3.7. Heart rate and blood pressure responses after the final ES exposure in Study 2

There was a significant increase in heart rate, systolic and diastolic blood pressure (Supplementary Figures S9A–C, respectively) and mean arterial pressure (Supplementary Figure S10A) in SDFA relative to NSFA and NSAP immediately after the light change. This increase in diastolic blood pressure was also relative to SDAP. There were a few other instances of increases in the blood pressure variables mostly in SDFA relative to one or more groups in the remaining 13.5 h period. There were significant increases in activity (Supplementary Figure S10B) mostly in NSAP relative to one or more groups during the entire monitoring period.

### 3.8. Cardiovascular function and dimensions measured using ultrasound after the final ES exposure in Study 2

**FIGURE 5:**
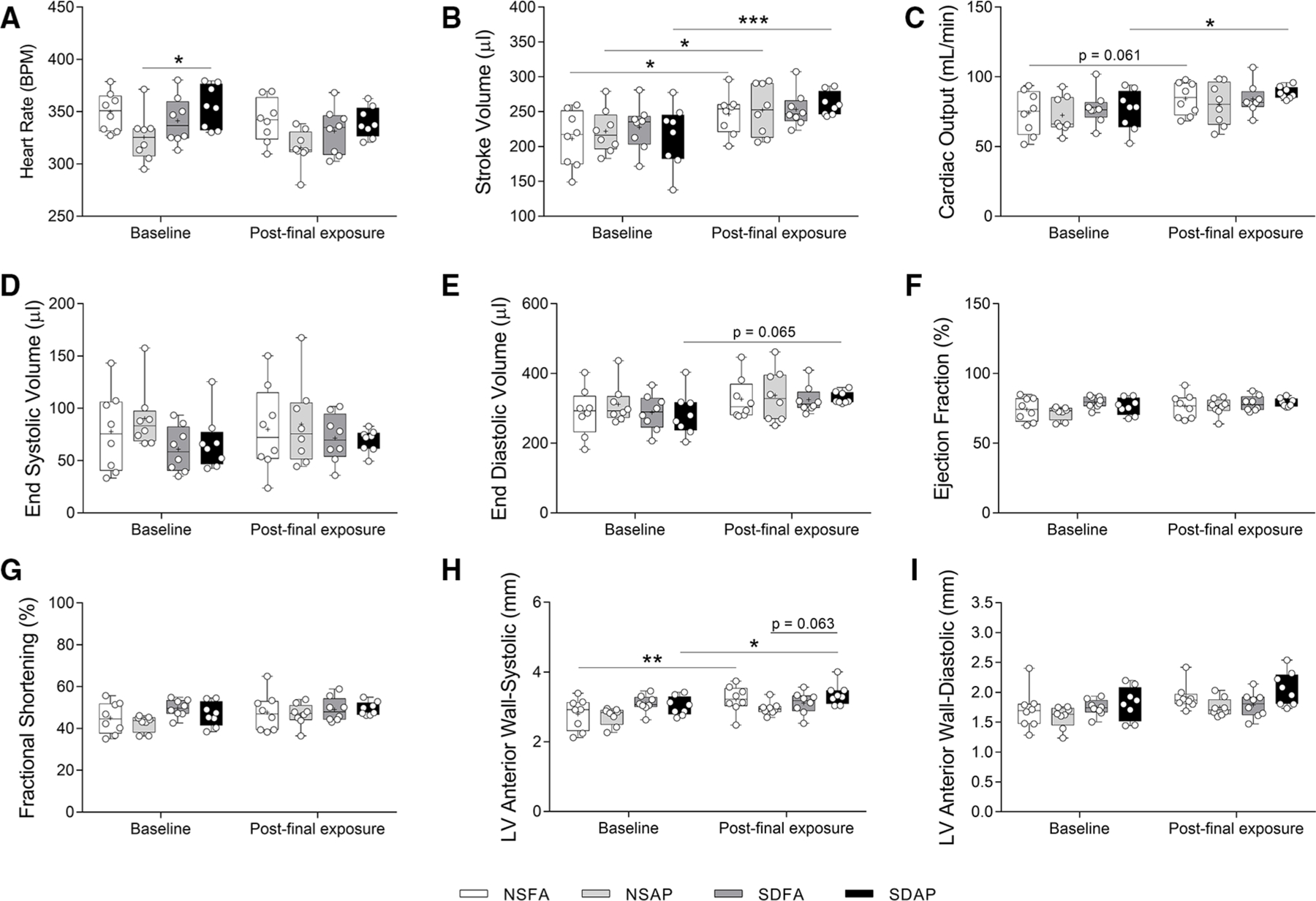
Cardiovascular function measured using cardiovascular ultrasound. Heart rate (**A**), stroke volume (**B**), cardiac output (**C**), end systolic volume (**D**), end diastolic volume (**E**), ejection fraction % (**F**), fractional shortening % (**G**), and left ventricular anterior wall thickness during systole (**H**) and diastole (**I**) were measured ~1 week before Day 1 and three days after the final day of sleep disruption and ES exposure. Data are reported using boxplots (*n* = 8). * - significant difference among groups (*p* < 0.05). ** - significant difference among groups (*p* = 0.002). *** - significant difference among groups (*p* = 0.001). *P*-values for tendencies towards significant changes are also indicated.

NSFA, NSAP, and SDAP all had significant increases in stroke volume three days after the final air pollution exposure relative to their corresponding values measured at baseline prior to sleep disruption and ES exposure, although SDAP had a higher level of significance (Figure 5B). Only SDAP had significantly greater cardiac output (Figure 5C) and a tendency towards greater end diastolic volume [*p* = 0.065; (Figure 5E)] at the final assessment relative to its corresponding values measured at baseline. SDAP also had tendencies towards increased left ventricular anterior wall thickness during both systole (*p* = 0.063; Figure 5H) after the final exposure relative to the baseline period (Figures 5H,I). There were no significant changes in heart rate (Figure 5A), end systolic volume (Figure 5D), ejection fraction % (Figure 5F), fractional shortening (Figure 5G), or left posterior wall thickness (Supplementary Figure S11) among any of the groups.

### 3.9. Gene expression in the hypothalamus after first and final exposure days in Study 2

Each group was contrasted with the two-factor control (NSFA) and significantly altered gene expression is reported (±1.25-fold, *p*-adjusted <0.05). One day after a single sleep disruption, SDFA had significant increases in *Per2, Crhr2,* and *Nr3c2* (Table 2). There were no changes in expression for NSAP or SDAP. There were no significant changes post-Day 8.

### 3.10. Gene expression in the heart after the first exposure day of Study 2

**FIGURE 6:**
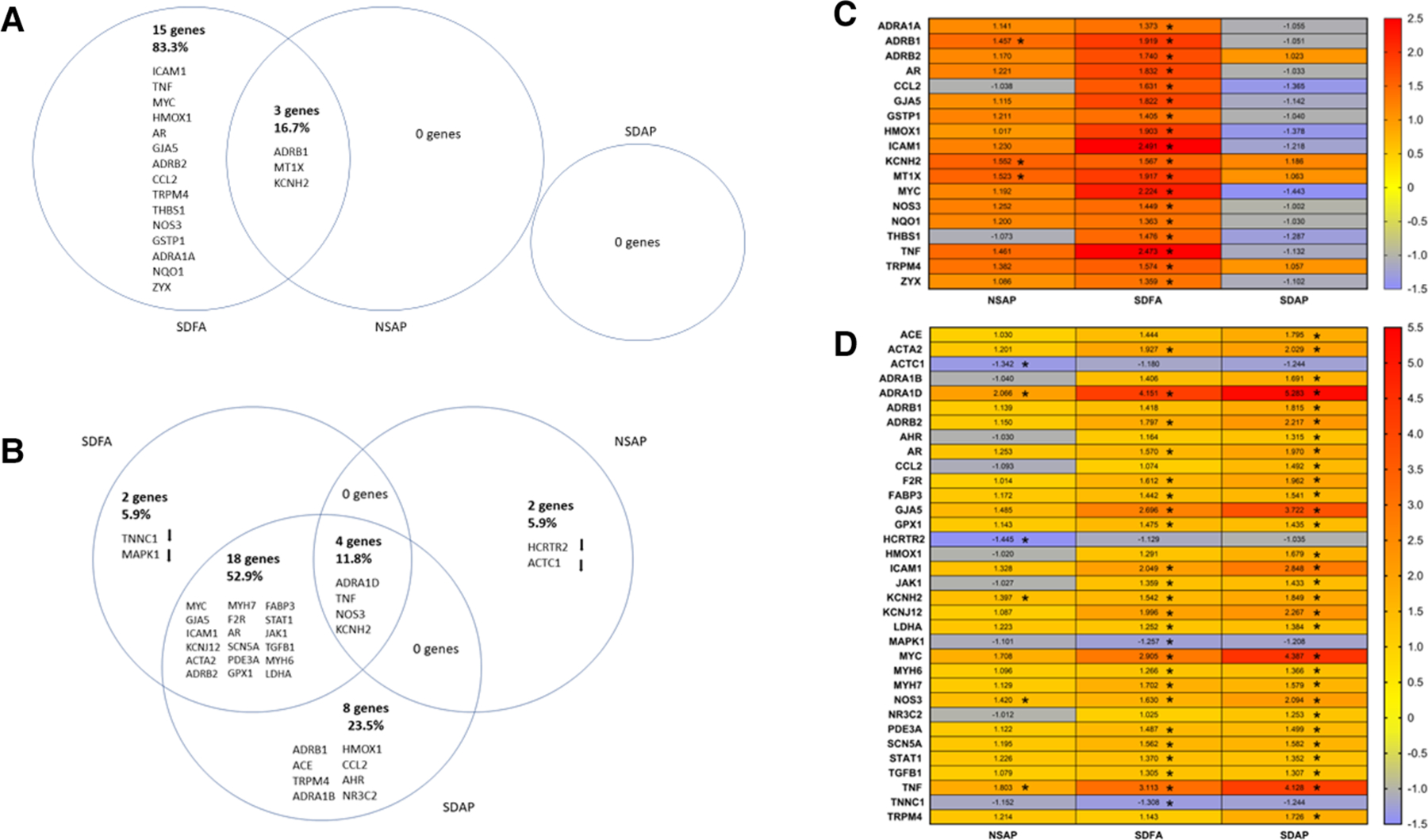
Venn diagrams show differences in gene expression in the left ventricle of the heart two days after Day 1 (**A**) and two days after the final day (**B**) of sleep disruption and ES exposure. The sleep-disrupted groups exposed to filtered air (SDFA) or eucalyptus smoke air pollution (SDAP) and the normal undisrupted group exposed to eucalyptus smoke air pollution (NSAP) are each compared to the normal undisrupted group exposed to filtered air (NSFA). The percentages of the total gene changes in all comparisons are provided for each comparison. Heat maps demonstrate the same comparisons relative to NSFA and indicate magnitude and direction of relative expression for Day 1 (**C**) and Day 8 (**D**). (*n* = 8). *=Fold change ≥ ± 1.25, *p*-adjusted <0.05.

Each group was contrasted with the two-factor control (NSFA) and significantly altered genes (±1.25-fold threshold, *p*-adj < *p*.05) are listed in Venn diagram format in Figure 6A. One day after a single sleep disruption, SDFA had significant increases in *Tnf, Mt1x, Icam1, Hmox1, Gstp1, Nqo1, Ccl2*, *Adra1a, Adrb1, Adrb2, Gja5, Kcnh2, Thbs1, Nos3, Trpm4, Myc, Ar,* and *Zyx* (18 genes in total) relative to the NSFA control (Figures 6A,C). Of these genes, 12 (*Tnf, Mt1x, Icam1, Hmox1, Ccl2*, *Adrb1, Adrb2, Gja5, Kcnh2, Trpm4, Myc, and Ar)* exceeded the ±1.50-fold threshold. NSAP had significant increases in *Mt1x, Adrb1*, and *Kcnh2,* increases that were shared with SDFA. Two of these genes (*Mt1x* and *Kcnh2)* exceeded the ±1.50-fold threshold. There were no significant increases in expression with SDAP on Day 1.

Because most of the significant changes that occurred with SDFA after one exposure day exceeded +/− 1.50-fold, gene ontology analysis in the three GO domains was performed at this cut-off. GO analysis revealed enrichment that was unique to SDFA across all three domains with lesser unique enrichment with NSAP (Supplementary Table S3).

### 3.11. Gene expression in the heart after the final exposure day of Study 2

One day after eight sleep disruptions and FA exposures, SDFA had significant increases in expression of *Adra1d, Tnf, Myc, Gja5, Icam1, Kcnj12, Acta2, Adrb2, Myh7, Nos3, F2r, Ar, Scn5a, Kcnh2, Pde3a, Gpx1, Fabp3, Stat1, Jak1, Tgfb1, Myh6, and Ldha* (22 genes in total) and decreases in *Tnnc1 and Mapk1*. Of these genes, 14 (*Adra1d, Tnf, Myc, Gja5, Icam1, Kcnj12, Acta2, Adrb2, Myh7, Nos3, F2r, Ar, Scn5a, Kcnh2*) exceeded the ±1.50-fold threshold. NSAP had significant increases in *Adra1d, Tnf, Nos3 and Kcnh2* and significant decreases in *Actc1* and *Hcrtr2*. Of these genes, 2 (*Adra1d, Tnf*) exceeded the ±1.50-fold threshold. SDAP had significant increases in 30 genes, including 18 that were shared with SDFA, 4 shared with NSAP and eight distinct genes including *Adra1b, Adrb1, Ace, Nr3c2, Ccl2, Hmox1, Ahr and Trpm4*. (Figures 6B, D). Of these genes, 20 (*Adra1d, Myc, Tnf, Gja5, Icam1, Kcnj12, Adrb2, Nos3, Acta2, Ar, F2r, Kcnh2, Adrb1, Ace, Trpm4, Adra1b, Hmox1, Scn5a, Myh7, Fabp3*) exceeded the ±1.50-fold threshold.

Because most of the significant changes that occurred with SDAP and SDFA after eight exposure days exceeded ±1.50-fold, GO analysis in the three GO domains was performed at this cut-off. GO analysis revealed enrichment that was unique to SDAP across all three domains with lesser unique enrichment with SDFA and NSAP (Table 3).

### 3.12. Lung and systemic factors after the first and final exposure days of Study 2

There were no significant differences among groups in any parameter measured in BAL fluid after the first sleep disruption/air pollution exposure. NSAP had a significant increase in hemoglobin in whole blood (Supplementary Table S4) relative to NSFA after one day of exposure. SDAP had significant increases in platelets relative to SDFA after the first exposure (Supplementary Table S4). Both SDFA and SDAP had significant increases in serum LDL relative to NSFA after one day of exposure (Supplementary Table S4). There were significant decreases in serum FABP and Myl3 in SDAP relative to NSFA after the first exposure. There were no significant differences among groups in any remaining systemic parameters after the first exposure day (Supplementary Table S4). SDAP had significantly fewer BAL macrophages than NSFA after the final sleep disruption/air pollution exposure (Supplementary Table S3). There was a significant increase in serum FFA in SDAP relative to NSAP. There was also a significant increase in serum ALT in SDAP relative to NSFA after the final sleep disruption/ES exposure. There were no significant differences in heart weight and body weight after the first or final exposure days (Supplementary Tables S4, S5).

## 4. Discussion

We adapted a model of sleep disruption in rats for the purposes of probing the potential for wildfire-related smoke inhalation to modify the cardiovascular responses associated with poor sleep. We established that gentle handling disrupted sleep, as indicated by reproducible awake-like locomotor activity and altered cardiovascular physiology. We then exposed sleep disrupted rats to ES, a complex combustion emission that we determined previously to be rich in polyaromatic hydrocarbons and volatile organic compounds and comparably more toxic than other biomass sources (32, 33). Exposure concentrations for this study were on par with particulate matter concentrations measured at monitoring stations during active fires in the Western USA [e.g., Pollock Pines, CA, USA (1.9 mg/m^3^), August 21, 2021; https://www.airnow.gov/]. ES exposure exaggerated heart rate and blood pressure responses and modified cardiac gene expression in sleep disrupted rats, suggesting that poor sleep related morbidity may be influenced by air pollution inhalation.

The study of sleeplessness in rats has been hampered by the lack of a widely accepted method to disrupt sleep. Rat models of sleep disruption vary considerably in technique and methods of validation and include, among other approaches, use of a slowly moving metal bar (34), which prevents rats from settling and resting, and the flowerpot method (35, 36), which is reliant on contact with water to awaken rats. Each method presents difficulties interpreting the translatability to human studies, however, gentle handling is a whole sleep disruption paradigm, as opposed to methods that target REM or non-REM alone. Gentle handling was chosen for its simplicity and the fact that the procedure by its very nature (i.e., gentle touch for 5 s once every 30 min during the sleep period) enables observation of the animal and confirmation of wakefulness. While gentle handling had been used in laboratory settings to reduce sleep, the impacts on cardiovascular function had not been characterized previously. In the present study, gentle handling increased activity, a surrogate metric for wakefulness, and several cardiovascular metrics including heart rate and blood pressure to levels observed during the active overnight period, providing evidence that gentle handling disrupted sleep and promoted wakefulness. In addition, the approach was consistent and reproducible given the similarity in responses across both the pilot and main study.

A single bout of sleep disruption and exposure to ES, but not either alone, increased heart rate and blood pressure shortly after exposure during the change from light to dark, a period during which rats transition from rest to wakefulness. This period is analogous to the transition to wakefulness in humans, one of the most clinically relevant periods for determination of cardiovascular risk as studies have shown that the early morning period is associated with peak risk for adverse cardiovascular events (37). The increase in blood pressure in rats in the present study is akin to morning blood pressure surge in humans, which is associated with elevated risk for stroke and heart attack (38), and such increased risk is elevated by both high daytime and nocturnal blood pressure (39, 40). The sleep disrupted group exposed to ES during the handling window in the final sleep disruption period also had significant increases in heart rate and blood pressure relative to either sleep disruption or ES exposure alone, again demonstrating that two factors combined worsened responses relative to either alone. During the subsequent post-handling period, however, the sleep disruption group exposed to FA had transition period increases in heart rate and blood pressure, responses that were not evident in any other group. These findings indicate that repeated sleep disruption alone over time also triggers increased “morning surge”-like risk, responses that were augmented by exposure to ES. The reason for the absence of such transition responses in the co-exposure group is not clear but may reflect some adaptation and/or biological variability to the stress associated with the transition to wakefulness. The demonstration of these changes primarily during the transition periods and while enduring the stress from handling suggests that the impacts from both factors combined may be insidious.

There was some evidence of systemic effects from sleep disruption as a single bout of sleep disruption in both sleep-disrupted groups increased serum LDL cholesterol, a factor widely linked to cardiovascular disease due to atherosclerosis risk (41). The immediacy of the functional responses after a single sleep disruption and exposure, however, suggests a limited role for these systemic changes, especially considering the absence of significant pulmonary and systemic inflammation. From measures of HRV immediately before the increase in heart rate and blood pressure during the Day 1 transition to the active period, there was evidence of increased sympathetic tone as indicated by a transient decrease in HRV (i.e., decreased SDNN) immediately before the transition to the active period only in the co-exposed group. This was followed by a sharp and sustained increase in parasympathetic tone akin to parasympathetic rebound, a phenomenon characterized by exaggerated vagal rebound after prolonged stress/sympathetic activity (42), as indicated by a significant increase in parasympathetic tone (i.e., increased SDNN, RMSSD) in the subsequent periods beginning at the transition to the active period. The downward overcorrection of the heart rate and blood pressure responses after their short-term increases provides support for overactive parasympathetic activity. The sustained parasympathetic response may have also been influenced by the prolonged handling stress during sleep disruption just a few hours earlier. These findings are consistent with the work of others which suggest autonomic modulation in responses to contemporaneous exposure to air pollution and sleep disruption. For example, Chuang et al. (43) determined that associations between PM_2.5_ concentration and indices of cardiac autonomic tone linked with decreased HRV were stronger during nightshift work, a period typically associated with perturbed sleep-wake cycles (43), and a study of wildland firefighters indicated similar autonomic indicators strongly correlated with job fatigue (44). Sympathetic tone increases heart rate by increasing activity of the sinus node (45, 46) and elevates blood pressure by increasing cardiac contractility and/or promoting vasoconstriction (47–49). Low HRV, reflecting increased sympathetic tone, is associated with an increased risk of cardiovascular morbidity and mortality (50), suggesting that air pollution exposure worsened sleep disruption-related cardiovascular risk. Although not assessed in the present study, this shift in autonomic tone may have been triggered by altered function of the baroreceptor, a principal mechanism for regulating blood pressure *via* sympathetic and parasympathetic modulation of heart rate and vascular smooth muscle function (51). Air pollution exposure and poor sleep have each been independently linked to altered baroreceptor sensitivity (50, 52). As for the functional effects during the final handling period, the mediators of these responses appear less clear as changes in HRV were few and not consistent. Long-term sleep disruption and ES exposure combined did, however, increase levels of the liver injury enzyme serum ALT, the elevated levels of which have been linked to exposure to particulate air pollution (53) and separately sleep disruption (54). The precise mechanisms driving the functional consequences from both short term and repeated co-exposures require further mechanistic studies.

Month-long co-exposure to sleep disruption and ES, but not exposure to either alone uncovered mild increases in stroke volume and cardiac output. These changes were accompanied by a tendency towards increased end diastolic volume suggesting increased preload, which increases in response to several factors (55) including increased aortic blood pressure. While the causes for these changes are unclear, they may be underpinned by persistent increases in blood pressure, which has been linked to left ventricular hypertrophy in the long-term due to cardiac pressure overload (56). Furthermore, chronic exposure to high levels of air pollution and nocturnal non-dipping have each been independently linked to cardiac remodeling, including hypertrophy (57–60).

A single sleep disruption in the FA group increased hypothalamic expression of *Per2*, a clock-related gene involved in circadian rhythm and regulation of sleep and wakefulness in mammals (61), relative to NSFA. These findings are consistent with previous findings that linked six-hour sleep deprivation in mice to increases in *Per2* in various brain regions (62), further validating the model of sleep disruption used in the present study. Changes in *Per2* expression were no longer evident after the eighth and final sleep disruption in either sleep disrupted group, perhaps indicating some adaptation to the rodent handling procedure. A single sleep disruption and FA exposure increased hypothalamic expression of key hormonal receptors including *Crhr2* and *Nr3c2*, which mediate stress and salt and water balance (63, 64), potentially indicating activation of the hypothalamic-pituitary-adrenal stress axis (65–67), although circulating markers of these processes were not measured in the present study.

A single sleep disruption alone modified expression of several key genes in the heart indicating a potential activation of processes that altered cardiac function. These included increases in genes related to (1) inflammation and oxidative stress (*Tnf*-α*, Mt1x, Icam1, Hmox1, Gstp1, NQO1* and *Ccl2*) (68–75), processes that have been extensively reported in cardiovascular disease (76, 77) and sleep-related disorders (78–81), (2) the adrenergic receptors *Adra1a, Adrb1 and Adrb2* involved in autonomic regulation, including the sleep-wake cycle (82), (3) the gap junction protein *Gja5*, and the potassium channel *Kcnh2*, both of which influence cardiac rhythm (83, 84), (4) the endothelial-related genes *Thbs1* and *Nos3*, both of which are notably altered in sleep disorders (85, 86), and (5) *Trpm4*, a mechanosensory channel expressed in the myocardium that plays a key role in mediating left ventricular hypertrophy induced by cardiac pressure overload (87). Gene ontology analysis of this data revealed enrichment in varied processes including apoptotic, chromosomal, adrenergic, and catecholamine activity, consistent with increased chronotropic and inotropic activity of the heart. By contrast, a single exposure to ES alone caused many fewer changes indicating a comparatively milder cardiac tissue response. One bout of sleep disruption and ES exposure caused no significant changes in cardiac gene expression, potentially pointing to the triggering of divergent and opposing biological responses, the precise nature of which is unclear. Eight sleep disruptions alone over the course of a month caused a greater increase in genes than a single disruption, increasing many of the same genes increased after Day 1 well as genes involved in adrenergic function [*Adra1d* (88)], inflammation and oxidative stress [*Gpx1, Tgfb1* (89, 90)], cell signaling [*Mapk1, Jak1, Stat1* (91–93)], platelet and vascular smooth muscle function [*F2r, Pde3a* (94, 95)], cytoskeletal and contractile markers [*Tnnc1, Fabp3, LDHA, Myh6* and *7*, and *Acta2* (95–99)], and ion channels involved in electrical conduction [*Scn5a* and *Kcnj12* (100, 101)]. These changes suggest that sleep disruption impacted normal functioning of cardiac tissue including excitation and contraction. By contrast, eight exposures to ES alone in undisrupted rats caused many fewer changes, increasing only four genes (*Nos3*, *Adra1d, Tnf* and *Kcnh2*) and decreasing expression of the cytoskeletal gene *Actc1* and the orexin-B receptor *Hcrtr2*, which is involved in sleep-wake regulation and has been demonstrated to be cardioprotective against ischemia-reperfusion injury in rats (102). Eight sleep disruptions and ES exposures combined over the course of a month caused an even greater increase in cardiac gene expression than sleep disruption alone. These increases included expression of eight distinct genes not increased with either alone including (1) adrenergic receptors (*Adra1b* and *Adrb1*), (2) regulators of electrolyte and water balance and blood pressure (*Ace* (103) and *Nr3c2* (67)), (3) inflammation and oxidative stress factors (*Ccl2* and *Hmox1*), (4) a regulator of responses to aromatic hydrocarbons [*Ahr* (104)], which are present in high levels in wildfire smoke (105–107), and (5) a stretch sensor (*Trpm4*). Go analysis revealed enrichment unique to SDAP after eight days of exposure for several processes including positive regulation of vasoconstriction, regulation of systemic arterial blood pressure by circulatory renin-angiotensin, and adrenergic receptor activity among other processes, consistent with the heart rate and blood pressure responses observed. These findings are aligned with previous findings that indicate that sleep disruption causes increases in adrenergic and renin-angiotensin system activity (108), responses made seemingly worse by co-exposure to air pollution. Collectively, these findings indicate that dual treatment caused a more pronounced myocardial response, perhaps emanating from exaggerated sympathetic activity and/or systemic inflammatory responses.

The present study is limited by several considerations. Rodent sleep is segmented differently than humans. Humans sleep nearly continuously during the night and are awake during a typical day (109, 110). Rodents, while nocturnal generally, do sleep some during their active period and are active during some of their resting period (111–113). This divergence in sleep behavior imparts difficulty in replicating human sleep disruption in rodent models. Rodents, however, do have periods which can be generalized as “active” and “resting.” This study also makes use of an inexact proxy of wakefulness (i.e., activity) which does not convey information regarding sleep architecture. The present study explored sleep disruption only in male rats. There is evidence that biological sex influences sleep patterns (114–116) and frequency and severity of cardiovascular disease incidence (117–121). Additionally, it is unknown if the strain of rat in the present study influenced results. Moreover, rats were not handled during their active period and thus the degree these changes may be due simply to stress that would be present during handling at any period is not known. Corticosterone analysis was limited by the timing of collection and additional differences may have been discerned if measured earlier or later than in this study. Furthermore, we did not measure corticosterone in Study 2 due to the lack of differences observed in Study 1. Finally, the genes analyzed in the presented study were a customized list of genes and therefore may include bias.

In conclusion, exposure to ES worsened sleep disruption-related cardiovascular pathophysiology. The exaggerated responses during the transition to wakefulness not only highlight the potential harmful impacts of just one night of poor sleep, but because of their transient nature, the possibility that these effects may be overlooked, potentially leading to underestimations of cardiovascular risk. The physiological impacts after long-term repeated sleep disruption and air pollution exposure were largely evident only during the final sleep disruption period and the transition to wakefulness and not while undisturbed, suggesting that the effects may be insidious and would perhaps manifest primarily in the presence of non-specific stressors of the cardiovascular system. The consequences from both short- and long-term sleep disruption are likely to be heightened in vulnerable groups including the elderly and individuals with pre-existing disease. The deleterious effects of poor sleep evident from these findings also suggest that sleep quality should be considered alongside well-established cardio-protective behaviors such as proper diet and regular exercise, particularly when considering vulnerability to environmental insult. Finally, as climate change is predicted to worsen both sleeplessness and air quality, the cumulative impacts of these factors may reduce the threshold for adverse outcomes in the years to come, increasing public health burden.

## Supplementary Material

s1

s2

s3

s4

s5

## Figures and Tables

**TABLE 1:** ES Exposure Constituent Concentrations.

	PM_2.5_ (mg/m^3^)	NO (ppm)	NO_2_ (ppm)	NOx (ppm)	CO_2_ (%)	CO (ppm)
Average	0.964	0.272	0.428	0.388	0.056	9.868
SD	0.330	0.068	0.007	0.044	0.011	2.432

These values represent the average of data from 18 different exposure runs unless otherwise noted. NO, NO2, and NOx, were measured during only 4 of the 18 runs and CO2 was measured during 16 of the 18 runs.

**Table 2:** Hypothalamic gene expression from respective groups vs. NSFA following Day 1.

GENE	DESCRIPTION	NSAP	SDFA	SDAP
Crhr2	corticotropin releasing hormone receptor 2	1.013391	1.33103*	1.126967
Nr3c2	nuclear receptor subfamily 2, group C, member 2	1.117983	1.29343*	1.118961
Per2	period circadian regulator 2	1.053306	1.32563*	1.25182

Values represent fold changes relative to two-factor control NSFA group. Asterisks indicate statistical significance (*±1.25 Fold change, *p*-adjusted <0.05). Only three genes were significantly different in any group. Corresponding non-significant fold changes for those genes are reported for the other two groups.

**TABLE 3:** GO analysis of cardiac gene expression data from respective groups vs. NSFA on Day 8.

	NSAP	SDFA	SDAP
Biological	regulation of nitric-oxide synthase activity (TNF, NOS3; *p* = 0.00336) negative regulation of potassium ion transport (NOS3, KCNH2; p = 0.00979) response to superoxide (TNF, NOS3; *p* = 0.01902) response to oxygen radical (TNF, NOS3; *p* = 0.01902) removal of superoxide radicals (TNF, NOS3; *p* = 0.01902)	protein-containing complex disassembly (TNF, MYC, ADRB2; *p* = 0.00263) positive regulation of cellular biosynthetic process (TNF, MYC, ICAM1, ACTA2, ADRB2, NOS3, F2R, AR; *p* = 0.00609) positive regulation of nucleobase-containing compound metabolic process (TNF, MYC, ACTA2, ADRB2, NOS3, F2R, AR; *p* = 0.00892) protein deubiquitination (MYC, ADRB2, AR; *p* = 0.00958) positive regulation of cysteine-type endopeptidase activity involved in apoptotic process (TNF, MYC, F2R; *p* = 0.00958)	positive regulation of vasoconstriction (GJA5, ICAM1, F2R, TRPM4, ADRA1B; *p* = 0.00416) positive regulation of cellular biosynthetic process (MYC, TNF, ICAM1, ADRB2, NOS3, ACTA2 AR, F2R, HMOX1, FABP3; *p* = 0.00657) regulation of systemic arterial blood pressure by circulatory renin-angiotensin (GJA5, F2R, ACE; *p* = 0.00824) protein-containing complex disassembly (MYC, TNF, ADRB2; *p* = 0.00824) glucose homeostasis (ICAM1, TRPM4, ADRA1B; *p* = 0.00824)
Cellular Component	plasma membrane part (ADRA1D, TNF, HCRTR2, NOS3, KCNH2; *p* = 0.04606) phagocytic cup (TNF; *p* = 0.06316) actomyosin, actin portion (ACTC1; *p* = 0.06316) inward rectifier potassium channel complex (KCNH2; *p* = 0.06316) membrane (KCNH2; *p* = 0.06316)	cell surface (TNF, ICAM1, F2R, SCN5A, KCNH2; *p* = 0.03091) membrane raft (TNF, ICAM1, NOS3, F2R, SCN5A; *p* = 0.03091) membrane region (TNF, ICAM1, NOS3, F2R, SCN5A; *p* = 0.03091) membrane microdomain (TNF, ICAM1, NOS3, F2R, SCN5A; *p* = 0.03091) plasma membrane (ADRA1D, TNF, GJA5, ICAM1, KCNJ12, ADRB2, NOS3, F2R, AR, SCN5A, KCNH2; *p* = 0.06314)	caveola (NOS3, F2R, ADRA1B, HMOX1, SCN5A; *p* = 0.00416). integral component of membrane (ADRA1D, TNF, GJA5, ICAM1, KCNJ12, ADRB2, F2R, KCNH2, ADRB1, ACE, TRPM4, ADRA1B, SCN5A; *p* = 0.01452). intrinsic component of membrane (ADRA1D, TNF, GJA5, ICAM1, KCNJ12, ADRB2, F2R, KCNH2, ADRB1, ACE, TRPM4, ADRA1B, SCN5A; *p* = 0.01452). integral component of plasma membrane (ADRA1D, TNF, GJA5, ICAM1, ADRB2, F2R, KCNH2, ADRB1, TRPM4, ADRA1B, SCN5A; *p* = 0.01815). intrinsic component of plasma membrane (ADRA1D, TNF, GJA5, ICAM1, ADRB2, F2R, KCNH2, ADRB1, TRPM4, ADRA1B, SCN5A; *p* = 0.01815).
Molecular Function	scaffold protein binding (NOS3, KCNH2; *p* = 0.03078) cadmium ion binding (NOS3; *p* = 0.06316) NADPH-hemoprotein reductase activity (NOS3; *p* = 0.12296) monooxygenase activity (NOS3; *p* = 0.12296) nitric-oxide synthase activity (NOS3; *p* = 0.12296)	inward rectifier potassium channel activity (KCNJ12, KCNH2; *p* = 0.02038) scaffold protein binding (NOS3, SCN5A, KCNH2; *p* = 0.02179) protein domain specific binding (GJA5, AR, SCN5A, KCNH2; *p* = 0.03775) voltage-gated potassium channel activity (KCNJ12, KCNH2; *p* = 0.05588) voltage-gated ion channel activity (KCNJ12, SCN5A, KCNH2; 0.06314)	adrenergic receptor activity (ADRA1D, ADRB2, ADRB1, ADRA1B; *p* = 0.01685). G protein-coupled amine receptor activity (ADRA1D, ADRB2, ADRB1, ADRA1B; *p* = 0.01685). protein domain specific binding (GJA5, AR, KCNH2, ADRB1, SCN5A; *p* = 0.0317). inward rectifier potassium channel activity (KCNJ12, KCNH2; *p* = 0.04255). scaffold protein binding (NOS3, KCNH2, SCN5A; *p* = 0.06114).

## Data Availability

All data are included as a supplementary Excel file. In addition, the results of all statistical tests used to support conclusions in this manuscript are included as a separate Excel file in the Supplementary Material.
