# Supplementary material for: Wildfire-related smoke inhalation worsens cardiovascular risk in sleep disrupted rats
Source: Front Environ Health. Author manuscript; Available in PMC 2024 May 30. (PMC10726696; doi:10.3389/fenvh.2023.1166918)

**Martin et al. Supplementary Material**

**Supplementary Table 1:** Experimental groups

| **Cohort 1: Telemetry** | |  |  |  | **Cohort 2: Early Necropsy** | | |  | **Cohort 3: Late Necropsy** | | |
| --- | --- | --- | --- | --- | --- | --- | --- | --- | --- | --- | --- |
|  | **Exposure** | **Measurements** | **Sample Size** |  | **Exposure** | **Measurements** | **Sample Size** |  | **Exposure** | **Measurements** | **Sample Size** |
| **Group 1** | **NSFA** | **Physiology Endpoints** | **7-8** |  | **Group 1** | **NSFA** | **8** |  | **Group 1** | **NSFA** | **8** |
| **Group 2** | **SDFA** | **Physiology Endpoints** | **7-8** |  | **Group 2** | **SDFA** | **8** |  | **Group 2** | **SDFA** | **8** |
| **Group 3** | **NSAP** | **Physiology Endpoints** | **7-8** |  | **Group 3** | **NSAP** | **8** |  | **Group 3** | **NSAP** | **8** |
| **Group 4** | **SDAP** | **Physiology Endpoints** | **7-8** |  | **Group 4** | **SDAP** | **8** |  | **Group 4** | **SDAP** | **8** |

**Supplementary Table 2:** HRV data imputation

| **Parameter** | **Missing & imputed data (%)** | |
| --- | --- | --- |
|  | **Day 1** | **Day 8** |
| **LF/HF** | **11.7** | **21** |
| **HF** | **11.7** | **21** |
| **LF** | **11.7** | **21** |
| **pNN50** | **1.5** | **3.5** |
| **RMSSD** | **1.5** | **3.5** |
| **SDNN** | **1.5** | **3.5** |

**Supplementary Table 3**: Gene ontology analysis of cardiac gene expression data from respective groups vs. NSFA on Day 1

|  | NSAP | SDFA | SDAP |
| --- | --- | --- | --- |
| Biological | - negative regulation of growth (MT1X, ADRB1; p = 0.02297) - regulation of heart rate by hormone (KCNH2; p = 0.03158) - cellular zinc ion homeostasis (MT1X; p = 0.03158) - detoxification of copper ion (MT1X; p = 0.03158) - response to erythropoietin (MT1X; p = 0.03158) | - protein-containing complex assembly (ICAM1, TNF, MYC, HMOX1, AR, GJA5, ADRB2, TRPM4; p = 0.00047) - protein-containing complex disassembly (TNF, MYC, ADRB2; p = 0.00159) - fat cell differentiation (TNF, ADRB1, ADRB2, TRPM4; p = 0.00437) - regulation of epithelial cell apoptotic process (ICAM1, TNF, HMOX1, CCL2; p = 0.00437) - protein deubiquitination (MYC, ADRB2, AR; p = 0.00589) | - no genes |
| Cellular Component | - perinuclear region of cytoplasm (KCNH2, MT1X; p = 0.02297) - Schaffer collateral - CA1 synapse (ADRB1; p = 0.03158) - inward rectifier potassium channel complex ((KCNH2; 0.03158) | - nuclear chromosome (MYC, AR; p = 0.04117) - chromatin (MYC, AR; p = 0.04117) - nuclear chromatin (MYC, AR; p = 0.04117) - chromosome (MYC, AR; p = 0.04117) - nucleolus (MYC, HMOX1; p = 0.04117) | - no genes |
| Molecular Function | - protein domain specific binding (KCNH2, ADRB1; p = 0.02850) - beta1-adrenergic receptor activity (ADRB1; p = 0.03158) - guanyl-nucleotide exchange factor activity (ADRB1; p = 0.03158) - Ras guanyl-nucleotide exchange factor activity (ADRB1; p = 0.03158) - neurotransmitter receptor activity (ADRB1; p = 0.03158) | - protein domain specific binding (ADRB1, AR, GJA5, KCNH2; p = 0.02094) - beta-adrenergic receptor activity (ADRB1, ADRB2; p = 0.04117) - epinephrine binding (ADRB1, ADRB2; p = 0.04117) - norepinephrine binding (ADRB1, ADRB2; p = 0.04117) - catecholamine binding (ADRB1, ADRB2; p = 0.04117) | - no genes |

**Supplementary Table 4**: BALF, whole blood, and serum factors after a single sleep disruption and smoke exposure.

| Group | Normal Sleep -  Filtered Air | Normal Sleep –  Air Pollution | Sleep Disrupted – Filtered Air | Sleep Disrupted –Air Pollution |
| --- | --- | --- | --- | --- |
| Body weight (g) | 561 ± 36 | 537 ± 35 | 538 ± 27 | 512 ± 23 |
| Heart wt/tibial length (mg/mm) | 23.90 ± 0.85 | 24.58 ± 2.07 | 24.05 ± 1.68 | 24.03 ± 2.65 |
| BALF Albumin (mg/L) | 16.92 ± 5.19 | 17.75 ± 5.95 | 14.27 ± 7.15 | 15.47 ± 7.56 |
| BALF Protein (mg/ml) | 90.41 ± 14.96 | 85.15 ± 20.82 | 91.52 ± 16.30 | 85.50 ± 26.78 |
| BALF GGT (U/I) | 6.01 ± 0.70 | 6.63 ± 1.01 | 5.85 ± 0.94 | 5.41 ± 0.92 |
| BALF LDH (U/I) | 29.98 ± 8.92 | 31.91 ± 12.14 | 29.65 ± 7.13 | 25.95 ± 5.19 |
| BALF NAG (U/I) | 10.19 ± 0.53 | 9.92 ± 0.37 | 10.00 ± 0.27 | 9.78 ± 0.45 |
| BALF Macrophages (cells/mI) | 58631 ± 20925 | 52251 ± 13593 | 66004 ± 18651 | 55672 ± 8785 |
| BALF Neutrophils (cells/mI) | 2052 ± 1118 | 2087 ± 1773 | 1342 ± 1430 | 1796 ± 2515 |
| BALF Lymphocytes (cells/mI) | 2858 ± 1580 | 2579 ± 1339 | 2328 ± 743 | 1418 ± 635 |
| BALF Total Cells (cells/mI) | 63541 ± 21637 | 56918 ± 14982 | 69674 ± 19811 | 58886 ± 11133 |
| Whole Blood WBC (K/ml) | 6.54 ± 1.62 | 6.10 ± 1.24 | 5.19 ± 1.79 | 6.72 ± 1.62 |
| Whole Blood RBC (M/ml) | 7.54 ± 0.49 | 7.71 ± 0.35 | 7.47 ± 0.41 | 7.52 ± 0.28 |
| Whole Blood HB (g/dL) | 15.16 ± 0.79 | 16.36 ± 0.54^a^ | 15.26 ± 1.04 | 15.86 ± 0.90 |
| Whole Blood HCT (%) | 42.80 ± 2.87 | 43.70 ± 1.55 | 42.28 ± 2.37 | 42.55 ± 1.84 |
| Whole Blood MCV (fL) | 56.81 ± 2.53 | 56.74 ± 1.76 | 56.61 ± 2.40 | 56.61 ± 1.57 |
| Whole Blood MCH (pg) | 20.14 ± 0.86 | 21.26 ± 0.92 | 20.45 ± 1.44 | 21.08 ± 0.74 |
| Whole Blood MCHC (g/dL) | 35.49 ± 1.71 | 37.45 ± 1.20 | 36.08 ± 1.05 | 37.29 ± 1.33 |
| Whole Blood RDW (%) | 15.44 ± 0.92 | 15.58 ± 0.95 | 15.58 ± 0.80 | 15.48 ± 0.29 |
| Whole Blood PLT (K/ml) | 985.0 ± 117.8 | 914.5 ± 73.15 | 880.3 ± 85.82 | 1001 ± 73.48^b^ |
| Whole Blood PCT (%) | 632.8 ± 89.66 | 609.3 ± 53.33 | 585.0 ± 58.08 | 669.3 ± 66.91 |
| Whole Blood MPV (fL) | 6.41 ± 0.22 | 6.66 ± 0.27 | 6.65 ± 0.22 | 6.68 ± 0.32 |
| Serum Total Chol. (mg/dl) | 43.21 ± 6.46 | 42.33 ± 4.10 | 42.89 ± 5.74 | 42.82 ± 6.07 |
| Serum LDL Chol. (mg/dl)) | 5.98 ± 1.01 | 6.98 ± 1.24 | 7.52 ± 1.05^a^ | 7.56 ± 1.06^a^ |
| Serum HDL Chol. (mg/dl) | 17.14 ± 2.49 | 17.08 ± 2.51 | 16.95 ± 2.27 | 16.57 ± 2.12 |
| Serum Triglycerides (mg/dl) | 93.75 ± 50.14 | 77.37 ± 26.22 | 81.74 ± 26.46 | 92.63 ± 37.87 |
| Serum FFA (mM) | 401.00 ± 232.90 | 297.60 ± 55.77 | 336.60 ± 151.60 | 306.70 ± 118.40 |
| Serum ACE (U/I) | 236.40 ± 27.79 | 232.90 ± 48.79 | 216.40 ± 30.45 | 241.70 ± 41.45 |
| Serum ALP (U/I) | 27.96 ± 5.21 | 25.60 ± 3.25 | 29.27 ± 2.74 | 25.79 ± 4.26 |
| Serum ALT (U/I) | 45.93 ± 11.46 | 45.48 ± 5.15 | 46.30 ± 8.24 | 54.25 ± 12.36 |
| Serum C3 (mg/dl) | 39.37 ± 2.45 | 40.00 ± 2.61 | 40.43 ± 2.79 | 40.87 ± 2.53 |
| Serum C4 (mg/dl) | 3.87 ± 0.82 | 3.83 ± 0.62 | 4.01 ± 0.35 | 3.92 ± 0.54 |
| Serum CK (U/I) | 435.70 ± 275.10 | 245.40 ± 105.80 | 272.00 ± 192.70 | 261.50 ± 164.00 |
| Serum FABP (pg/ml) | 11.26 ± 8.83 | 5.30 ± 2.09 | 5.71 ± 2.91 | 4.75 ± 4.30^a^ |
| Serum Myl3 (pg/ml) | 3.05 ± 4.63 | 1.15 ± 1.17 | 0.79 ± 0.63 | 0.60 ± 0.49^a^ |

Values represent means ± standard deviation (n= 7 or 8/group). BALF = bronchoalveolar lavage fluid; GGT- gamma glutamyl transferase; LDH = lactate dehydrogenase; NAG = N-acetyl glucosaminidase; WBC = white blood cell count, RBC = red blood cell count, HB = hemoglobin; HCT = hematocrit; CV = mean corpuscular volume (); MCH = mean corpuscular hemoglobin; MCHC = mean corpuscular hemoglobin concentration; RDW (%) – red blood distribution width; PLT = platelet count; PCT – plateletcrit; MPV = mean platelet volume. Chol. = cholesterol; LDL = low density lipoprotein; HDL = high density lipoprotein; FFA – free fatty acids; ACE = angiotensin converting enzyme; ALP = alkaline phosphatase; ALT = alanine aminotransferase; C3 = complement component C3; C4 = complement component C4; CK = creatine kinase; FABP = fatty acid binding protein; Myl3 = myosin light chain 3. a – significantly different from Normal-Filtered Air; b – p = 0.053 vs. Sleep Disrupted-Filtered Air; Notes: 1) For heart weight data, group NSAP has only 7 values per group; 2) For BALF GGT, NAG and Protein data, groups NSAP and SDFA have only 7 values per group; 3) For BALF LDH and MIA data, group NSAP has only 7 values per group.

**Supplementary Table 5:** BALF, whole blood, and serum factors after eight sleep disruptions and smoke exposures.

| Group | Normal Sleep- Filtered Air | Normal Sleep – Air Pollution | Sleep Disrupted – Filtered Air | Sleep Disrupted –Air Pollution |
| --- | --- | --- | --- | --- |
| Body weight (g) | 555 ± 35 | 550 ± 43 | 533 ± 18 | 567 ± 49 |
| Heart wt/tibial length (mg/mm) | 23.72 ± 1.46 | 23.27 ± 1.50 | 24.02 ± 1.63 | 24.42 ± 2.52 |
| BALF Albumin (mg/L) | 17.85 ± 4.25 | 18.39 ± 6.32 | 20.88 ± 10.39 | 17.09 ± 3.83 |
| BALF Protein (mg/ml) | 88.86 ± 11.65 | 92.80 ± 20.33 | 99.63 ± 27.33 | 92.39 ± 19.41 |
| BALF GGT (U/I) | 5.42 ± 0.55 | 5.72 ± 1.01 | 6.22 ± 1.61 | 5.57 ± 0.58 |
| BALF LDH (U/I) | 24.34 ± 6.15 | 26.84 ± 4.76 | 29.57 ± 5.05 | 27.01 ± 4.02 |
| BALF NAG (U/I) | 10.05 ± 0.24 | 10.10 ± 1.13 | 10.37 ± 0.69 | 9.64 ± 0.40 |
| BALF Macrophages (cells/mI) | 59135 ± 12695 | 53525 ± 20131 | 53014 ± 13107 | 40020 ± 6696^a^ |
| BALF Neutrophils (cells/mI) | 5370 ± 5529 | 3087 ± 3459 | 5921 ± 5665 | 10141 ± 9353 |
| BALF Lymphocytes (cells/mI) | 2493 ± 1409 | 3671 ± 4347 | 4579 ± 4252 | 3866 ± 2135 |
| BALF Total Cells (cells/mI) | 66998 ± 10538 | 60283 ± 24784 | 63514 ± 14627 | 54026 ± 12379 |
| Whole Blood WBC (K/ml) | 6.32 ± 1.16 | 6.24 ± 1.38 | 7.72 ± 2.37 | 7.10 ± 0.92 |
| Whole Blood RBC (M/ml) | 7.66 ± 0.34 | 7.80 ± 0.22 | 7.66 ± 0.38 | 7.49 ± 0.33 |
| Whole Blood HB (g/dL) | 16.43 ± 0.68 | 16.08 ± 0.37 | 16.09 ± 0.91 | 15.65 ± 0.45 |
| Whole Blood HCT (%) | 42.33 ± 1.99 | 42.85 ± 0.91 | 41.59 ± 2.11 | 40.88 ± 1.13 |
| Whole Blood MCV (fL) | 55.35 ± 3.37 | 54.91 ± 1.51 | 54.36 ± 2.48 | 54.66 ± 2.45 |
| Whole Blood MCH (pg) | 21.48 ± 0.93 | 20.60 ± 0.61 | 21.01 ± 1.17 | 20.94 ± 1.05 |
| Whole Blood MCHC (g/dL) | 38.83 ± 0.94 | 37.53 ± 0.87 | 38.69 ± 1.39 | 38.31 ± 1.35 |
| Whole Blood RDW (%) | 15.10 ± 0.48 | 15.36 ± 0.54 | 15.23 ± 0.58 | 15.65 ± 0.85 |
| Whole Blood PLT (K/ml) | 952.00 ± 114.50 | 945.30 ± 66.89 | 928.90 ± 75.98 | 931.10 ± 105.7 |
| Whole Blood PCT (%) | 624.80 ± 68.78 | 591.90 ± 48.54 | 585.80 ± 46.55 | 603.40 ± 88.92 |
| Whole Blood MPV (fL) | 6.58 ± 0.30 | 6.26 ± 0.23 | 6.31 ± 0.32 | 6.46 ± 0.30 |
| Serum Total Chol. (mg/dl) | 46.13 ± 4.55 | 46.42 ± 4.82 | 46.56 ± 5.15 | 48.79 ± 10.91 |
| Serum LDL Chol. (mg/dl)) | 9.36 ± 1.50 | 8.03 ± 1.25 | 8.39 ± 1.35 | 7.91 ± 1.21 |
| Serum HDL Chol. (mg/dl) | 18.44 ± 1.60 | 17.42 ± 2.15 | 17.99 ± 1.44 | 19.12 ± 4.12 |
| Serum Triglycerides (mg/dl) | 86.77 ± 28.46 | 97.28 ± 38.69 | 89.42 ± 31.18 | 121.60 ± 74.08 |
| Serum FFA (mM) | 352.40 ± 97.38 | 316.30 ± 82.10 | 390.00 ± 165.50 | 479.60 ± 127.70^b^ |
| Serum ACE (U/I) | 252.10 ± 48.05 | 229.00 ± 35.65 | 250.90 ± 16.09 | 258.70 ± 37.78 |
| Serum ALP (U/I) | 24.92 ± 3.85 | 25.47 ± 5.00 | 32.63 ± 14.90 | 31.33 ± 5.54 |
| Serum ALT (U/I) | 43.28 ± 10.47 | 50.67 ± 10.68 | 47.84 ± 5.79 | 55.79 ± 7.71^a^ |
| Serum C3 (mg/dl) | 40.90 ± 3.47 | 40.78 ± 3.84 | 38.22 ± 1.95 | 39.75 ± 3.52 |
| Serum C4 (mg/dl) | 3.87 ± 0.82 | 3.83 ± 0.62 | 4.01 ± 0.35 | 3.92 ± 0.54 |
| Serum CK (U/I) | 176.20 ± 62.84 | 188.70 ± 90.04 | 169.10 ± 76.75 | 202.30 ± 55.37 |
| Serum FABP (pg/ml) | 5.69 ± 7.63 | 4.79 ± 3.28 | 4.25 ± 2.11 | 3.85 ± 1.97 |
| Serum Myl3 (pg/ml) | 0.49 ± 0.47 | 0.82 ± 1.65 | 0.76 ± 0.67 | 0.32 ± 0.29 |

Values represent means ± standard deviation (n= 7 or 8/group). BALF = bronchoalveolar lavage fluid; GGT- gamma glutamyl transferase; LDH = lactate dehydrogenase; NAG = N-acetyl glucosaminidase; WBC = white blood cell count, RBC = red blood cell count, HB = hemoglobin; HCT = hematocrit; MCV = mean corpuscular volume; MCH = mean corpuscular hemoglobin; MCHC = mean corpuscular hemoglobin concentration; RDW (%) – red blood distribution width; PLT = platelet count; PCT – plateletcrit; MPV = mean platelet volume. Chol. = cholesterol; LDL = low density lipoprotein; HDL = high density lipoprotein; FFA – free fatty acids; ACE = angiotensin converting enzyme; ALP = alkaline phosphatase; ALT = alanine aminotransferase; C3 = complement component C3; C4 = complement component C4; CK = creatine kinase; FABP = fatty acid binding protein; Myl3 = myosin light chain 3. a – significantly different from Normal-Filtered Air. b - significantly different from Normal Sleep-Air Pollution. Notes: For Myl3 data, groups NSFA and NSAP have only 7 values per group.

**Supplementary Figure Legends**

**Figure S1**: Cardiovascular responses and activity measured during rodent handling in Study 1 pilot. Relative activity (A), heart rate (B), systolic (C) and diastolic (D) blood pressure and mean arterial pressure (E) were recorded during the entire 5-hour handling period. Data represent averages over the entire handling period of data recorded (n=8) every 10 min in normal rats and sleep-disrupted rats. *- significant difference among groups (p < 0.05).

**Figure S2**: Plasma corticosterone immediately and one day after rodent handling in Study 1 pilot. Data represent plasma levels in naïve rats and in rats immediately and one day after the 5-hour handling period (n=8).

**Figure S3:** Cardiovascular responses and activity averaged over 5 hours during rodent handling on Day 1 in Study 2. Relative activity (A, F), heart rate (B, G), systolic (C, H) and diastolic (D, I) blood pressure, and mean arterial pressure (E, J) were recorded during the entire 5-hour handling period. Data (n=8) in Panels A thru E represent averages when all normal rats are grouped together (n=16) and all sleep-disrupted rats (n=15) are grouped together while data in Panels F thru J represent averages when each of the normal and sleep disrupted groups were each split into two groups (Group 1 and 2), which eventually became the filtered air and air pollution groups.

**Figure S4:** Cardiovascular responses and activity measured every 10 min over 5 hours during rodent handling on Day 1 in Study 2. Relative activity (A), heart rate (B), systolic (C) and diastolic (D) blood pressure and mean arterial pressure (E) were recorded during the entire 5-hour handling period. Data (n=8) represent average values every 10 min when the normal and sleep disrupted groups were each split into two groups (Group 1 and 2), which eventually became the filtered air and air pollution groups.

**Figure S5:** Cardiovascular responses before and after the transition from light to dark one day after exposure to eucalyptus smoke or filtered air. Heart rate (A), systolic (B) and diastolic (C) blood pressure, mean arterial pressure (D) and activity (E) were recorded while rats were in their home cages approximately 90 min before and 70 min after the light change. Data represent averages of 3 minutes of data (n=7-8) recorded every 10 min in normal rats exposed to filtered air (NSFA) or eucalyptus smoke air pollution (NSAP) and sleep-disrupted rats exposed to filtered air (SDFA) or eucalyptus smoke air pollution (SDAP).

**Figure S6:** Frequency domain heart rate variability responses before and after the transition from light to dark immediately after exposure to eucalyptus smoke or filtered on Day 1. Low frequency domain (LF; A), high frequency domain (HF; B) and the ration of LF over HF (LF/HF; C) were recorded while rats were in their home cages approximately 30 min before and 30 min after the light change. Data represent averages of 3 minutes of data recorded every 10 min in normal rats exposed to filtered air (NSFA) or eucalyptus smoke air pollution (NSAP) and sleep-disrupted rats exposed to filtered air (SDFA) or eucalyptus smoke air pollution (SDAP). a - significantly different from itself during the immediately preceding time period (p < 0.05). c – significantly different from NSFA within time period (p<0.05). f – significantly different than SDAP (p<0.05) within time period. (n=7-8).

**Figure S7**: Time domain heart rate variability responses for Day 8 Handling window. Root mean square of the standard deviation of the normal–normal RR interval (RMSSD; A), standard deviation of the normal–normal RR interval (SDNN; B) and the percent of adjacent normal RR intervals differing by ≥ 50 ms (pNN50; C) were recorded while rats were in their home cages during the entire 5-hour handling window. Data represent averages of 3 minutes of data (n=7-8) recorded every 10 min in normal rats exposed to filtered air (NSFA) or eucalyptus smoke air pollution (NSAP) and sleep-disrupted rats exposed to filtered air (SDFA) or eucalyptus smoke air pollution (SDAP). a - significantly different from itself during the immediately preceding time period (p < 0.05). b – significantly different than all other groups within time period (p<0.05). c – significantly different from NSFA within time period (p<0.05). d – significantly different than NSAP within time period (p<0.05). e – significantly different than SDFA (p<0.05) within time period. f – significantly different than SDAP (p<0.05) within time period. p-values for tendencies towards significant changes are also indicated.

**Figure S8**: Frequency domain heart rate variability responses for Day 8 Handling window. Low frequency domain (LF; A), high frequency domain (HF; B) and the ration of LF over HF (LF/HF; C) were recorded while rats were in their home cages during the entire 5-hour handling window. Data represent averages of 3 minutes of data (n=7-8) recorded every 10 min in normal rats exposed to filtered air (NSFA) or eucalyptus smoke air pollution (NSAP) and sleep-disrupted rats exposed to filtered air (SDFA) or eucalyptus smoke air pollution (SDAP a - significantly different from itself during the immediately preceding time period (p < 0.05). c – significantly different from NSFA within time period (p<0.05). p-values for tendencies towards significant changes are also indicated.

**Figure S9:** Cardiovascular responses following rodent handling on Day 8. These panels including heart rate (A), and systolic (B) and diastolic (C) blood pressure represent data recorded immediately following the return of the animals to their home cages and extend thru the entire lights off period. Data represent averages of 3 minutes of data (n=7-8) recorded every 10 min in normal rats exposed to filtered air (NSFA) or eucalyptus smoke air pollution (NSAP) and sleep-disrupted rats exposed to filtered air (SDFA) or eucalyptus smoke air pollution (SDAP). a – significantly different than NSFA (p < 0.05). b – significantly different than NSAP (p < 0.05). c - significantly different than SDAP (p < 0.05. d – significantly different than SDFA (p < 0.05).

**Figure S10:** Cardiovascular responses and activity following rodent handling on Day 8. These panels including mean arterial blood pressure (A) and relative activity (B) represent data recorded immediately following the return of the animals to their home cages and extend thru the entire lights off period. Data represent averages of 3 minutes of data (n=7-8) recorded every 10 min in normal rats exposed to filtered air (NSFA) or eucalyptus smoke air pollution (NSAP) and sleep-disrupted rats exposed to filtered air (SDFA) or eucalyptus smoke air pollution (SDAP). a – significantly different than NSFA (p < 0.05). b – significantly different than NSAP (p < 0.05). c - significantly different than SDAP (p < 0.05. d – significantly different than SDFA (p < 0.05).

**Figure S11:** Left ventricular posterior wall thickness measured using cardiovascular ultrasound. Left ventricular posterior wall thickness during systole (A) and diastole (B) were measured ~1 week before Day 1 and three days after the final day of sleep disruption and eucalyptus smoke exposure. Data are reported using boxplots (n = 8). p-values for tendencies towards significant changes are indicated.

**Supplemental Figures**

**Figure S1**


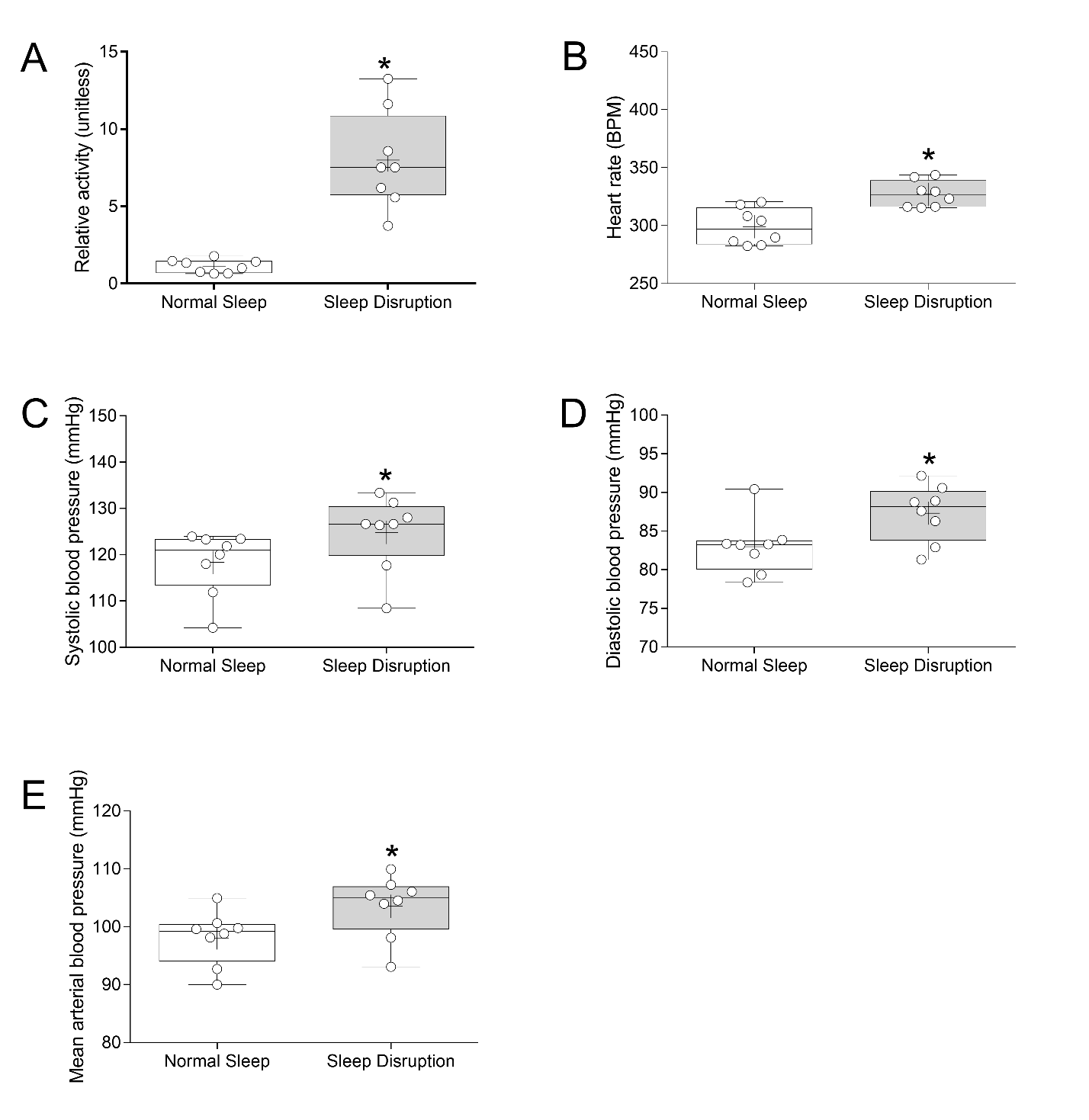


**Figure S2**


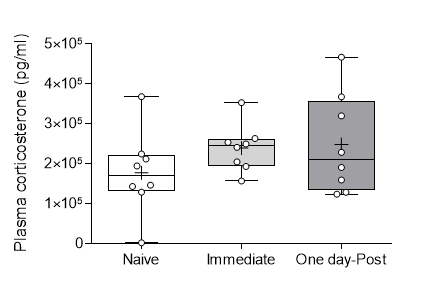


**Figure S3**


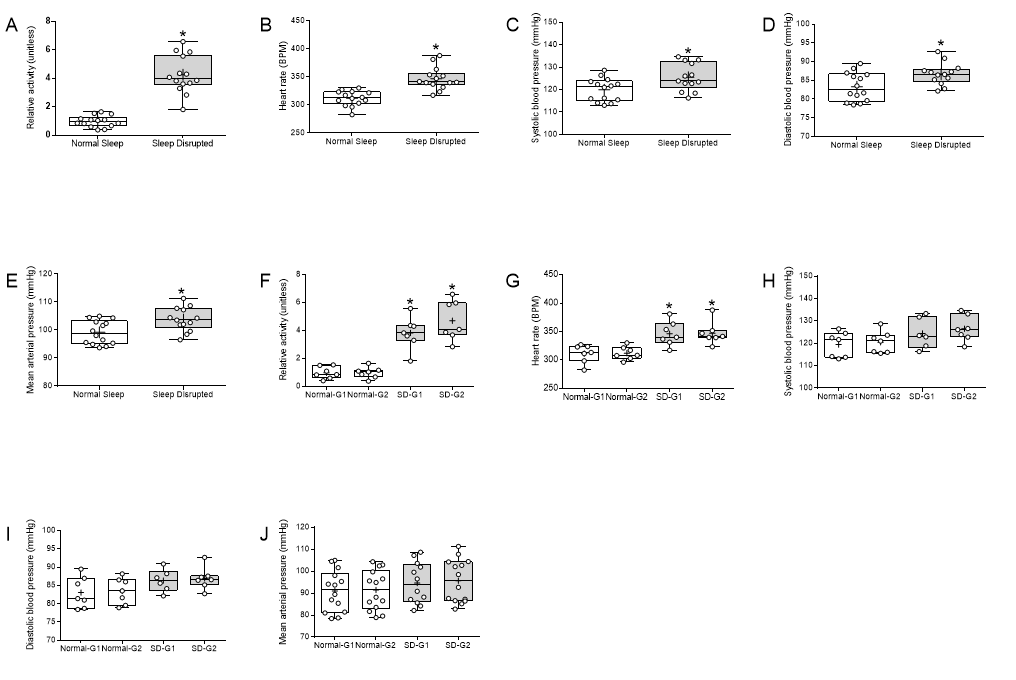


**Figure S4**


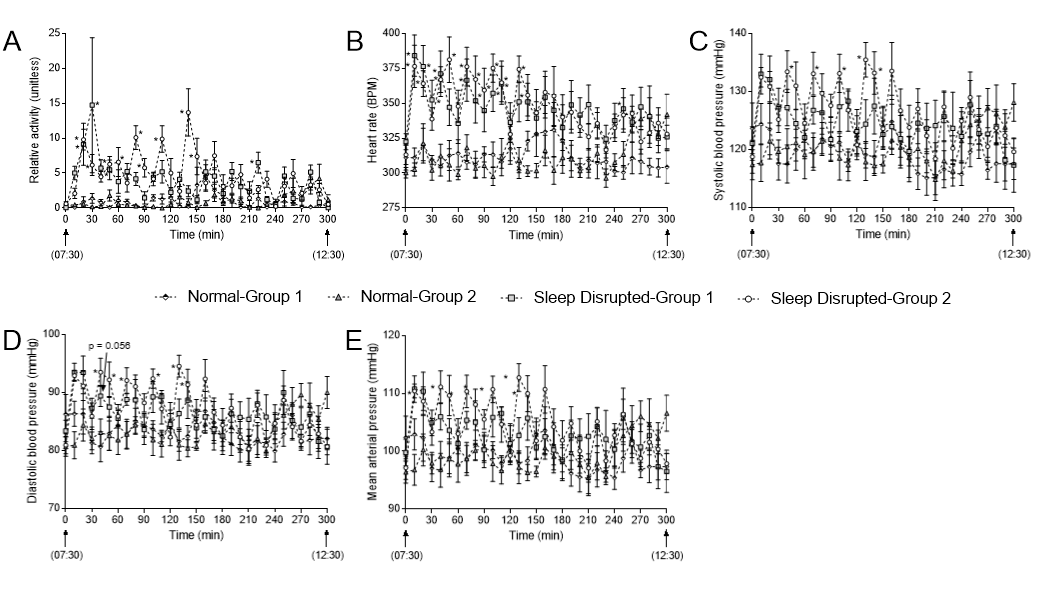


**Figure S5**


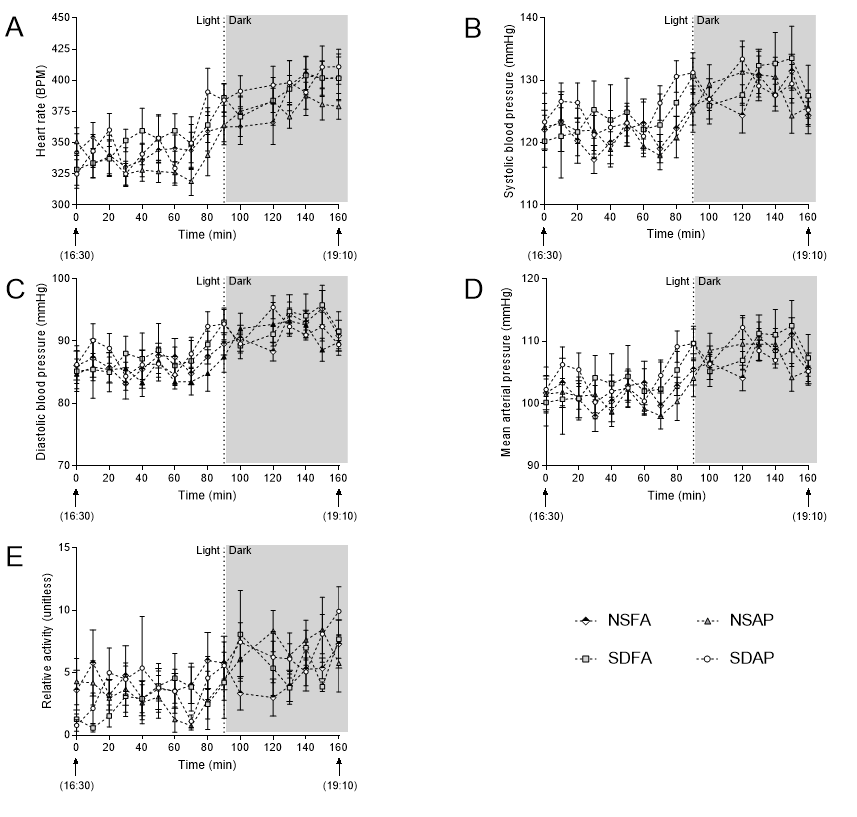


**Figure S6**


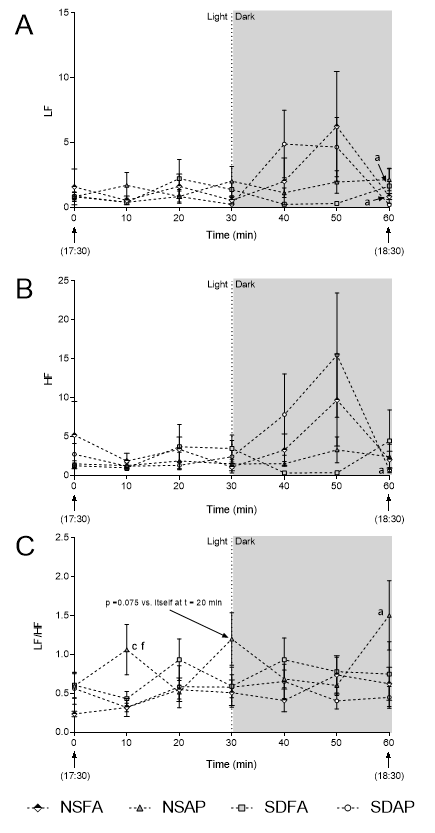


**Figure S7**


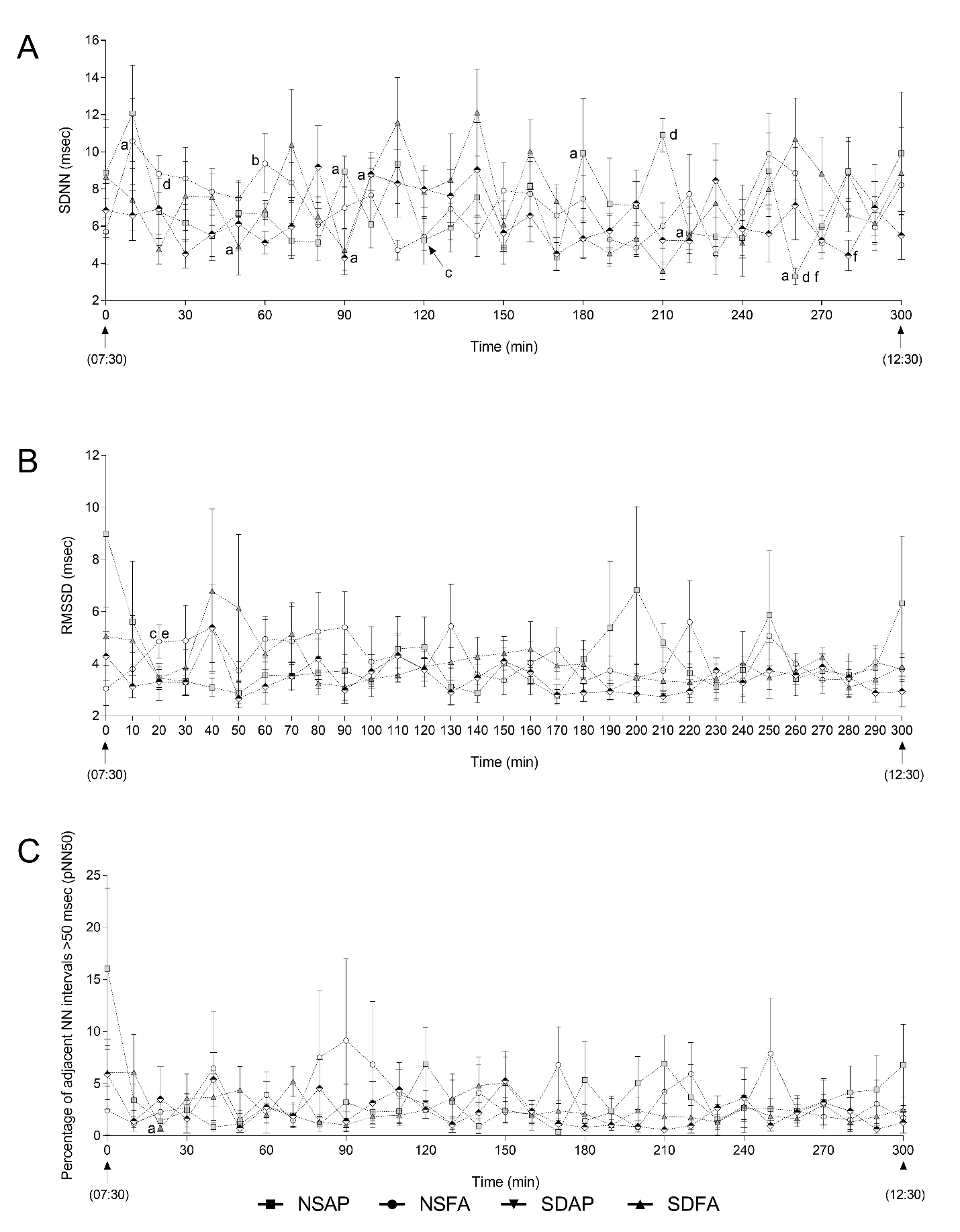


**Figure S8**

**
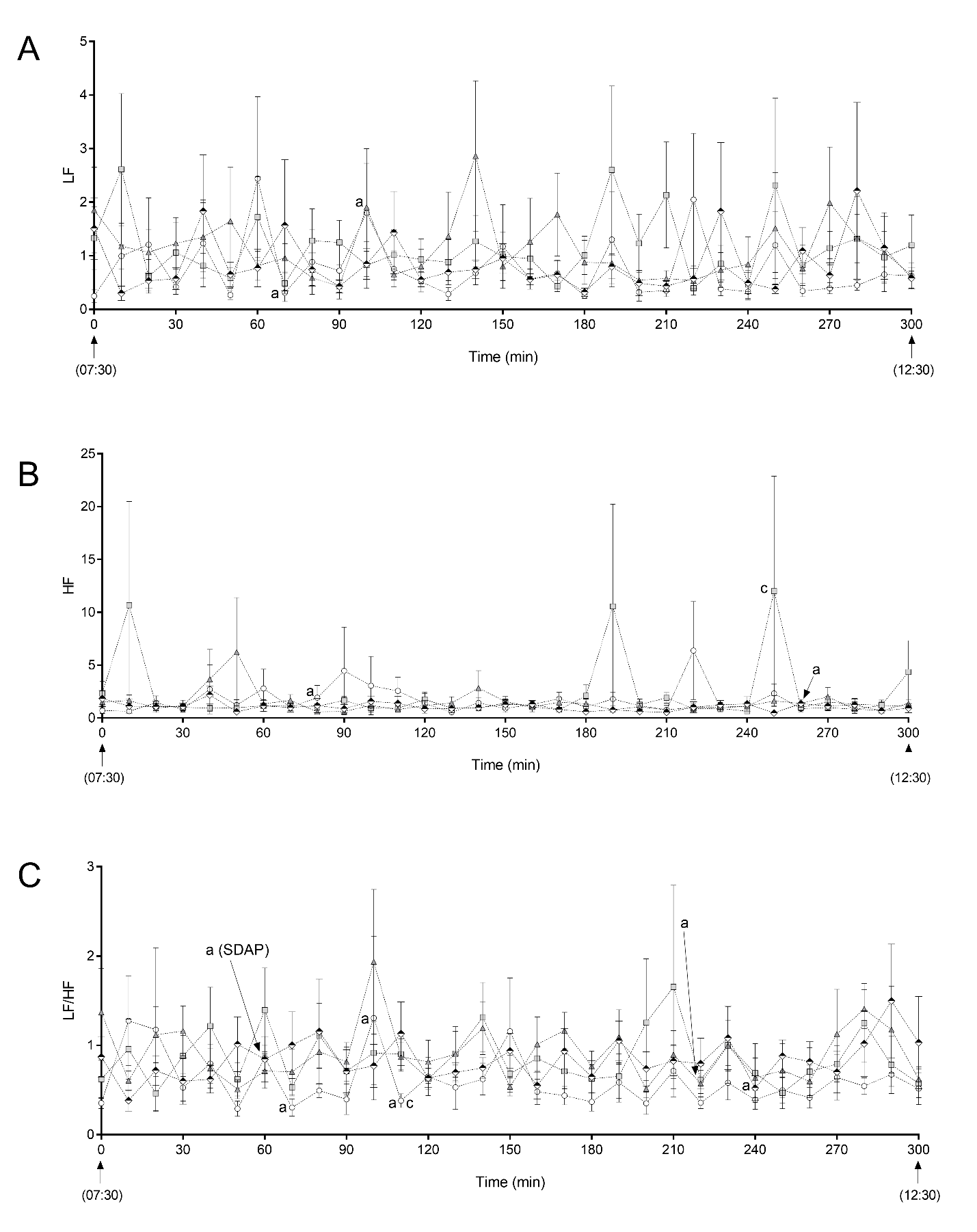
**

**Figure S9**
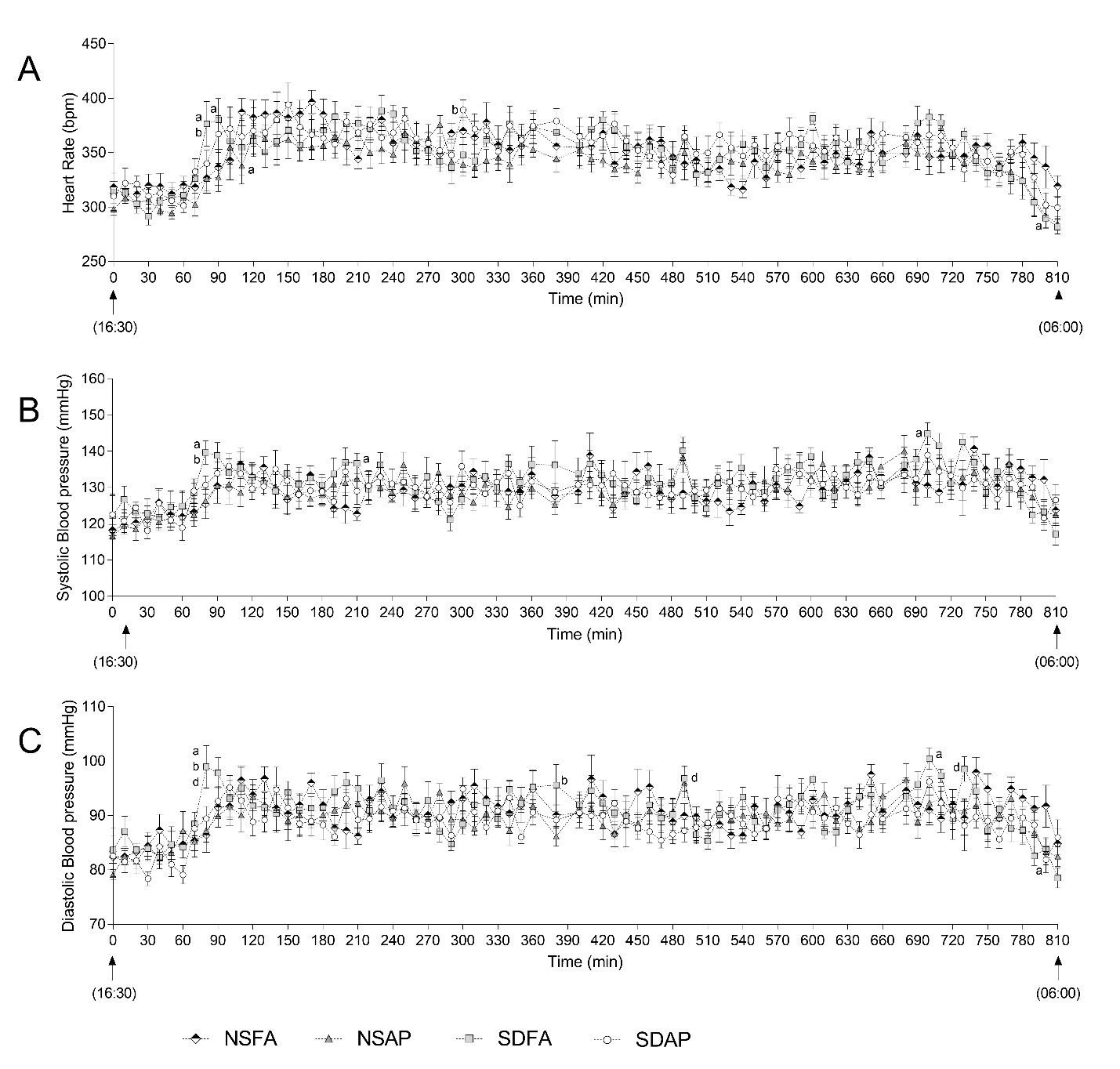


**Figure S10**


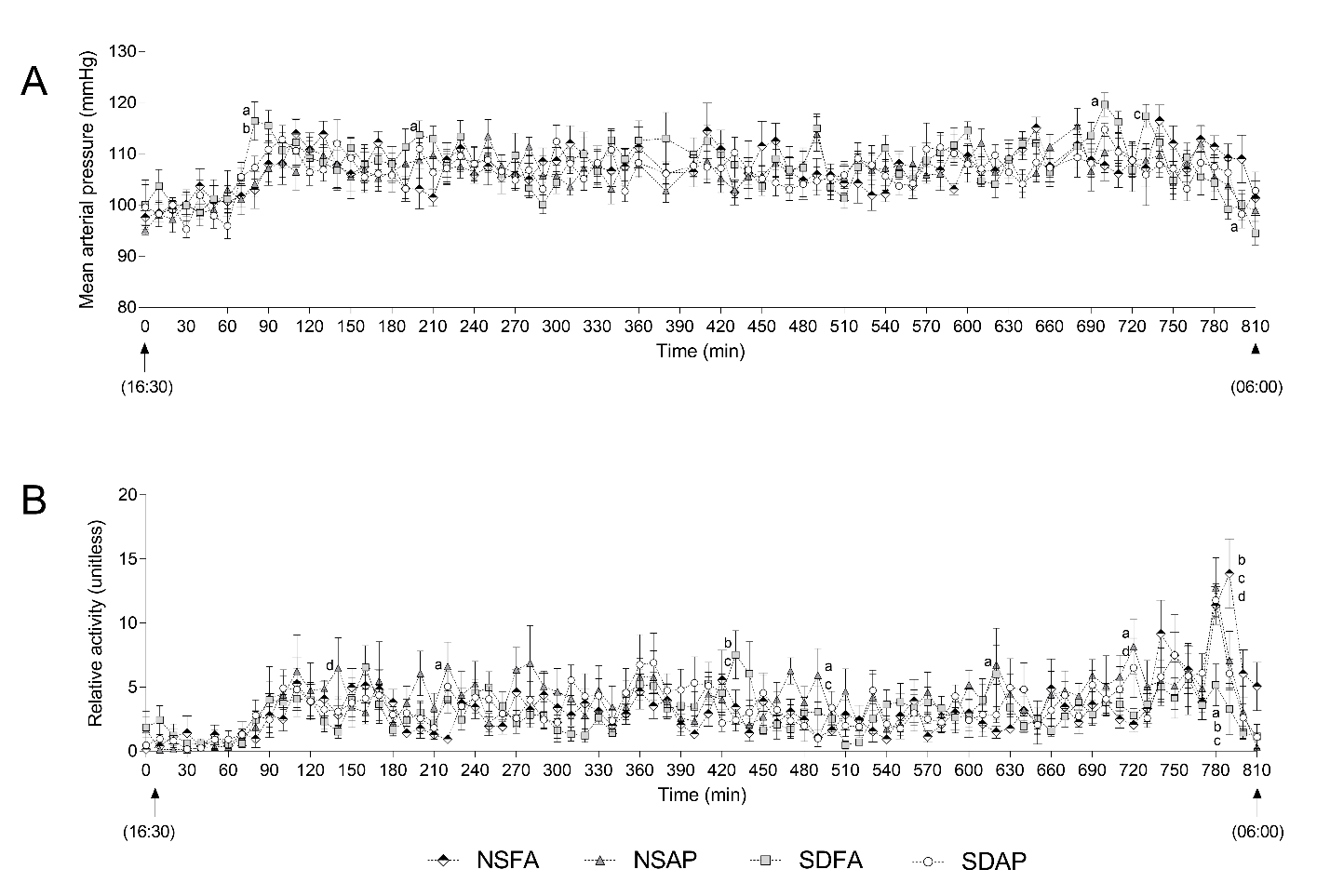


**Figure S11**

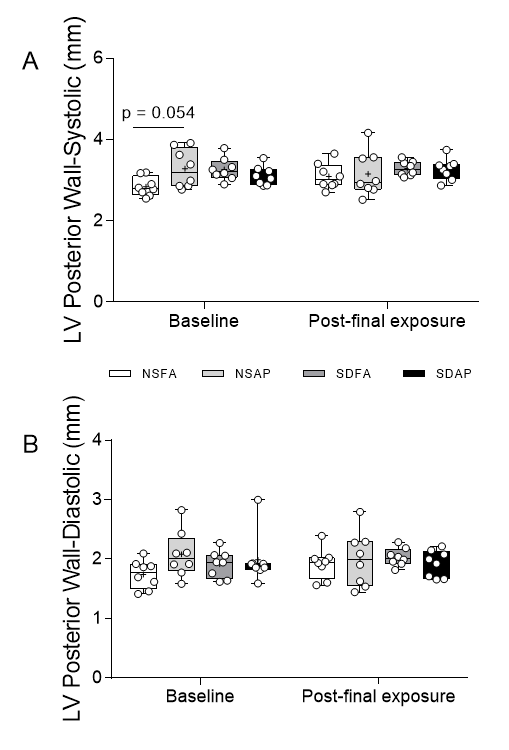

Supplement: s5 [file NIHMS1941972-supplement-s5.docx]
